# Ir/Rh-catalyzed hydrogenation of squalene: access to stereoregular squalane

**DOI:** 10.1039/d6ra05192h

**Published:** 2026-09-07

**Authors:** Teresa Iovine, Christian Ehm, Umesh I. Kaluarachchige Don, Vincenzo Busico, Adam S. Veige, Roberta Cipullo

**Affiliations:** a Dipartimento di Scienze Chimiche, Università di Napoli Federico II 80126 Napoli Italy christian.ehm@unina.it rcipullo@unina.it; b Department of Chemistry, University of Florida, Center for Catalysis P.O. Box 117200 Gainesville Florida 32611 USA veige@chem.ufl.edu; c DPI P.O. Box 902 5600 AX Eindhoven the Netherlands

## Abstract

Catalytic stereoselective hydrogenation of unfunctionalized polyenes remains a major challenge because multiple stereogenic centers must be generated in the absence of directing groups. Here we investigate squalene, a flexible hydrocarbon containing six isolated trisubstituted double bonds, as a demanding substrate for assessing stereochemical bias in the complete hydrogenation of an unfunctionalized polyene. Evaluation of Rh- and Ir-based homogeneous catalysts identified one Ir–P,N complex that promotes complete conversion to squalane while inducing a highly non-statistical diastereomeric distribution, reaching up to 90% d.e. The stereochemical outcome was quantified directly by ^13^C NMR spectroscopy of crude reaction mixtures, without derivatization or chromatographic separation. Buried-volume analysis was used to compare the steric environment of the selective Ir catalyst with representative non-selective systems, providing a qualitative interpretation of the observed behaviour. To our knowledge, this work provides the first catalytic access to stereoregular squalane and establishes a practical NMR-based approach to evaluate stereochemical bias in hydrogenated isoprenoid hydrocarbons.

## Abstract 2

An Ir–P,N catalyst enables complete stereoselective hydrogenation of squalene to stereoregular squalane with 90% d.e.

## Graphical abstract

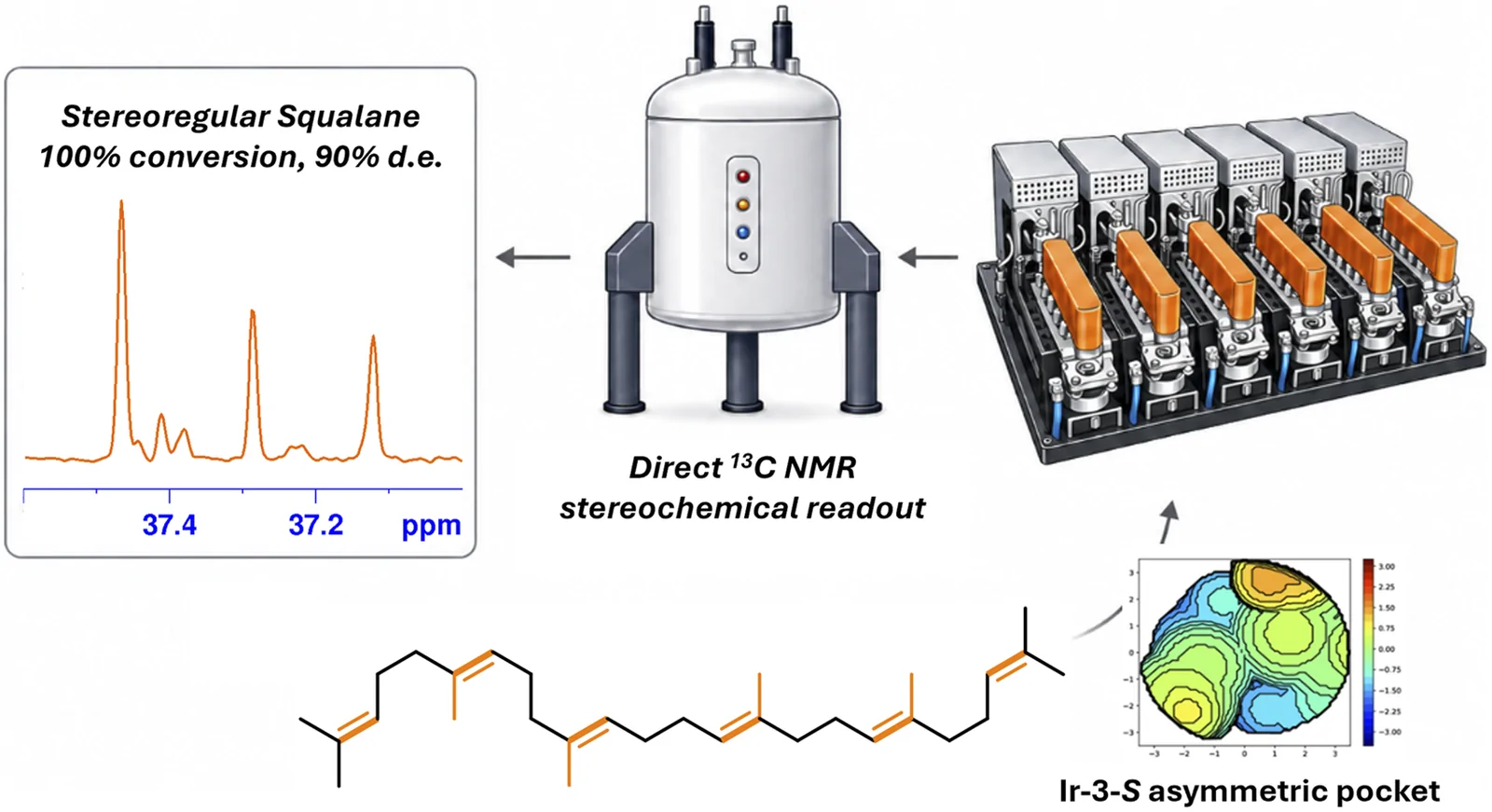

## Introduction

Catalytic asymmetric hydrogenation of C=C, C=O, and C=N double bonds is a powerful and atom-economical strategy for the synthesis of optically active molecules used in pharmaceuticals, agrochemicals, and fine chemicals.^1–15^ While highly efficient catalytic systems have been developed for substrates bearing coordinating functional groups, the stereoselective hydrogenation of unfunctionalized olefins remains a major challenge because of the absence of directing interactions and the subtle interplay between catalyst structure and stereochemical outcome.^16–19^

**Fig. 1:**
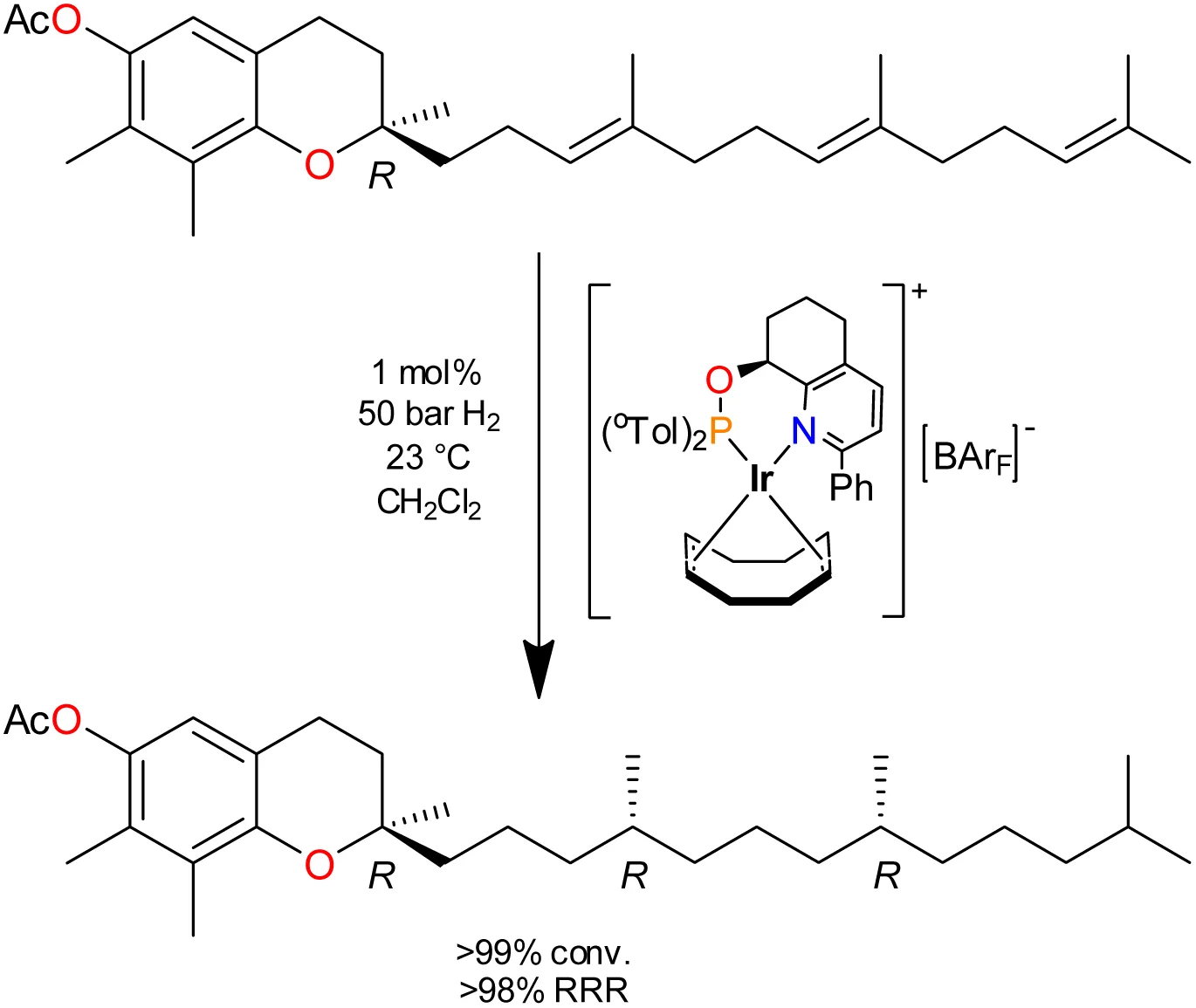
Enantioselective hydrogenation of γ-tocotrienyl acetate by Pfaltz. Adapted from ref. 19.

Among unfunctionalized olefins, alkyl-substituted alkenes are particularly difficult to hydrogenate selectively, with methyl-substituted double bonds representing one of the most demanding classes of substrates.^19^ Chiral iridium complexes derived from Crabtree-type catalysts, especially Ir–P,N systems,^20^ have provided some of the most successful solutions to this problem, enabling the asymmetric hydrogenation of di-, tri-, and tetrasubstituted unfunctionalized olefins.^16–23^ In a landmark contribution, Pfaltz *et al.* reported the stereoselective hydrogenation of γ-tocotrienyl acetate using an Ir–P,N catalyst, affording the *RRR* isomer with excellent stereocontrol (Fig. 1).^19^

Polyenes containing multiple isolated alkyl-substituted double bonds represent a further level of complexity. In these substrates, several stereogenic centers are generated in a single transformation, and the final product distribution reflects the cumulative outcome of multiple hydrogenation events.^24^ Thus, even when complete hydrogenation is achieved, the key question remains whether the catalyst can impose a measurable stereochemical bias throughout the entire polyene framework. This is especially challenging for flexible hydrocarbon substrates, where stereochemical control must arise without assistance from coordinating functional groups.

A further obstacle is analytical. For substrates bearing a single prochiral double bond, the stereochemical outcome can often be determined by established chromatographic methods, sometimes after derivatization.^25–28^ For polyenes, however, the number of possible stereoisomers increases rapidly, making product analysis substantially more demanding. In the case of γ-tocotrienyl acetate, for example, which contains three isolated C=C bonds in the side chain, determination of the isomeric composition required post-functionalization followed by one hour GC analysis.^19^ Direct and rapid characterization methods capable of evaluating stereochemical bias in complex hydrogenated hydrocarbon mixtures would therefore be particularly useful.

Herein, we address this challenge using squalene as a demanding substrate for stereoselective polyene hydrogenation. Squalene is a flexible hydrocarbon containing six isolated trisubstituted double bonds and no coordinating functional groups. Its complete hydrogenation generates squalane, a fully saturated isoprenoid hydrocarbon containing multiple stereogenic centers.

We evaluated a focused series of known homogeneous Ir- and Rh-based hydrogenation catalysts, benchmarked against Pd/C, under standardized miniaturized hydrogenation conditions. Hydrogenation efficiency and stereochemical outcome were rapidly evaluated directly from crude reaction mixtures by ^1^H and ^13^C NMR spectroscopy, avoiding derivatization or chromatographic separation. Among the catalysts examined, a single Ir–P,N complex achieved complete conversion to squalane while inducing a highly non-statistical diastereomeric distribution, reaching up to 90% diastereomeric excess. Buried-volume analysis was used to compare the steric environment of the selective Ir catalyst with representative non-selective systems, providing a qualitative interpretation of the observed behaviour. To the best of our knowledge, this represents the first catalytic access to stereoregular squalane.

## Results and discussion

### Substrate and catalytic systems

**Fig. 2:**
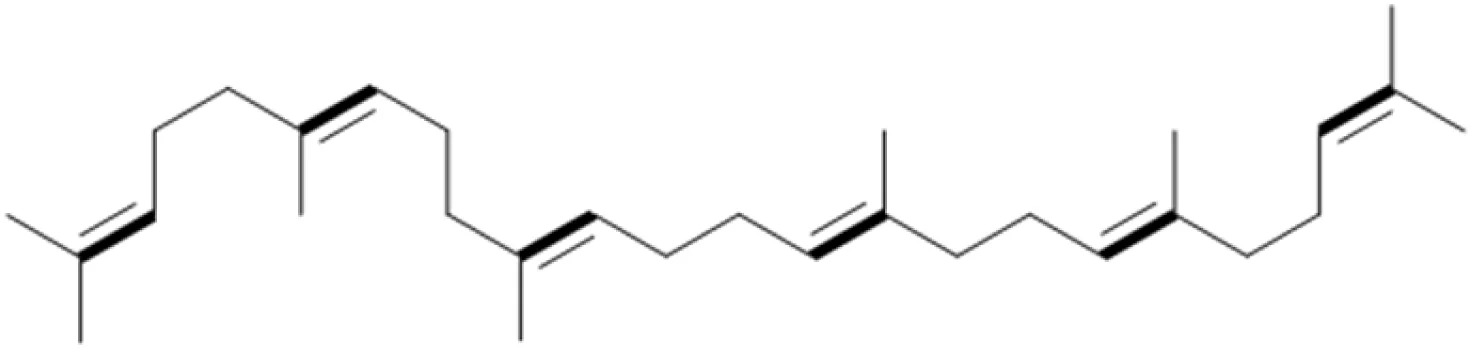
Chemical structure of squalene.

The terpenoid squalene, SQE, (6*E*,10*E*,14*E*,18*E*)-2,6,10,15,19,23-hexamethyltetracosa-2,6,10,14,18,22-hexaene (Fig. 2), was selected as a model substrate for stereoselective hydrogenation of unfunctionalized polyenes. Squalene is a naturally occurring hydrocarbon widely used in nutraceutical and cosmetic applications; however, its six isolated trisubstituted double bonds make it susceptible to oxidative degradation, leading to undesired by-products such as epoxides and alcohols.^29–31^ The four internal double bonds are *trans*-configured and prochiral, making SQE a demanding substrate for catalyst-controlled stereochemical induction.

Hydrogenation of SQE affords squalane, SQA, a fully saturated isoprenoid hydrocarbon with improved oxidative stability and broad use in cosmetic, pharmaceutical, and nutraceutical formulations, particularly as an emollient and lipid-based carrier.^32,33^ Previous catalytic routes to squalane have mainly employed heterogeneous metal catalysts, including Pd-based systems operating under mild or solvent-free conditions.^32,34^ From a catalytic perspective, however, controlling the stereochemical distribution of the resulting squalane remains particularly challenging.

The flexible 24-carbon backbone of SQE contains six chemically similar alkyl-substituted double bonds and no coordinating functional group; therefore, any stereochemical bias must arise from catalyst-controlled discrimination between weakly differentiated hydrocarbon substituents. For this reason, SQE provides a stringent test of both hydrogenation efficiency and stereochemical control in the absence of directing interactions.

**Fig. 3:**
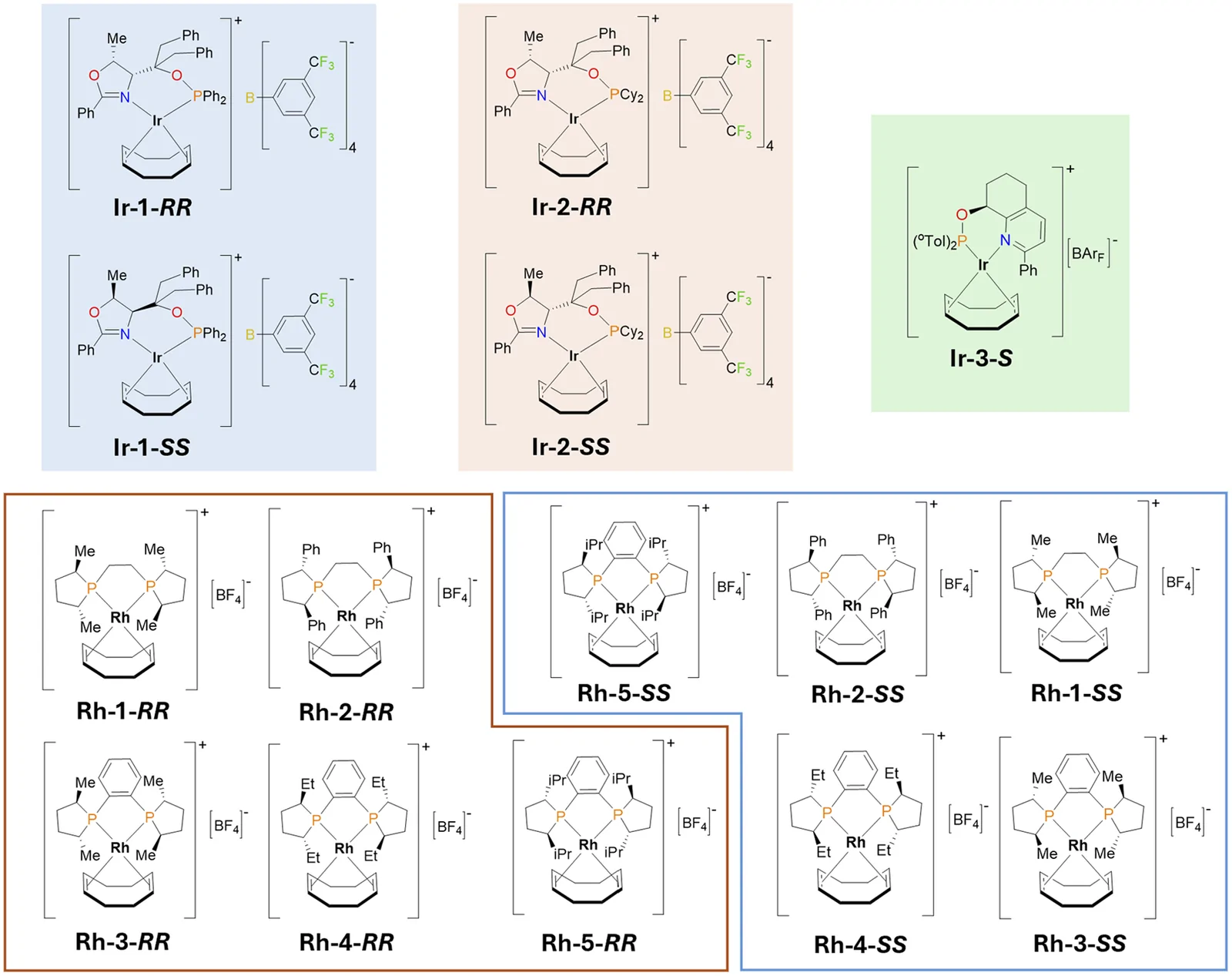
Screened hydrogenation homogeneous catalysts.

A focused library of homogeneous hydrogenation catalysts was selected to probe how metal identity and ligand architecture affect both hydrogenation efficiency and stereochemical induction in SQE hydrogenation. The catalyst library comprised five Ir-based complexes structurally related to the Pfaltz-type Ir–P,N systems (Fig. 3, top) and ten Rh-based complexes corresponding to five enantiomeric pairs (Fig. 3, bottom). The heterogeneous catalyst Pd/C was included as a non-selective benchmark.

### Catalyst screening and hydrogenation efficiency

Hydrogenation experiments were performed at least in duplicate using a Freeslate (former Symyx) Parallel Pressure Reactor (PPR) platform consisting of 48 reaction cells arrayed in six 8-cell modules and originally optimized for high-throughput studies of olefin polymerization catalysts.^35–41^ All catalysts were evaluated under identical experimental conditions (*i.e.*, *T* = 50 °C, *p*(H_2_) = 200 psi, 1.2 mol% catalyst loading relative to double bonds). The experimental procedure is reported in detail in the SI and in the Experimental section.

**Fig. 4:**
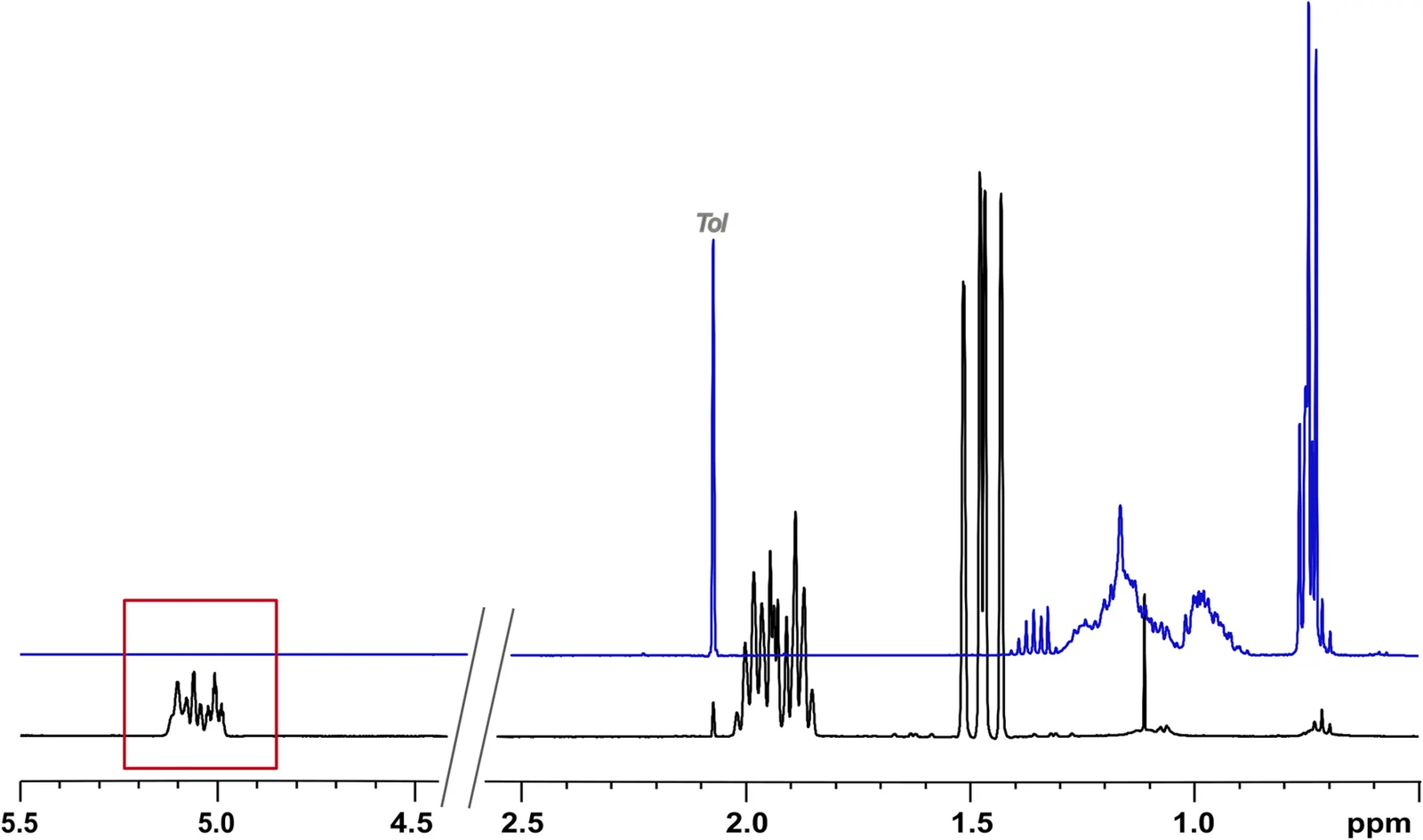
Overlay of the ^1^H NMR spectra of SQE (in black) and SQA (in blue) obtained with catalysts Ir-3-*S*.

**Fig. 5:**
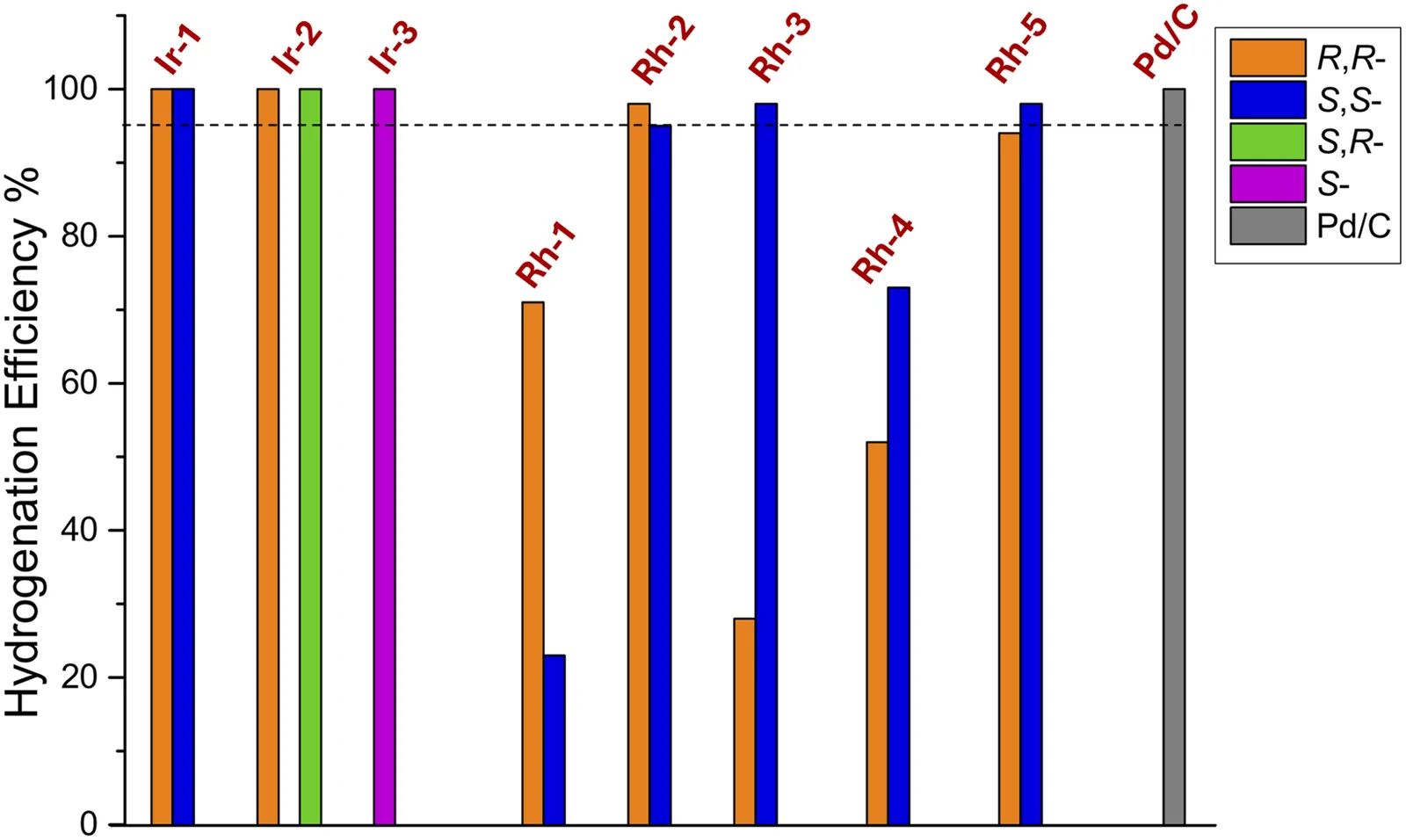
Hydrogenation efficiencies of the screened catalysts at 1.2 mol% catalyst loading.

A practical advantage of this protocol is that product work-up or purification is not required prior to analysis. Aliquots of the crude reaction mixtures were transferred directly to NMR tubes, enabling rapid assessment of hydrogenation efficiency by ^1^H NMR spectroscopy. Conversion was determined by integration of the olefinic region at 4.9–5.2 ppm relative to the full aliphatic/olefinic spectral region at 0.7–5.2 ppm. Representative ^1^H NMR spectra (acquisition time, 2 min) of SQE (in black) and SQA (in blue) obtained with catalyst Ir-3-*S* are shown in Fig. 4. The hydrogenation efficiency of all screened catalysts is reported as a percentage of hydrogenated SQE double bonds in Fig. 5 (see also SI).

All Ir-based complexes are competent hydrogenation catalysts under the employed reaction conditions, delivering complete conversion to SQA, similar to Pd/C. In contrast, the rhodium-based catalysts displayed lower and more variable activity, with only five out of ten complexes reaching hydrogenation efficiencies between 94% and 98%. Although the catalyst set is rather limited, no clear trend in the performance of the Rh-based catalysts toward SQE hydrogenation emerges. In most cases, the *R*,*R* enantiomer was more active than the corresponding *S*,*S* form, although Rh-1 showed the opposite behaviour. Changing the bridging moiety within the chelating ligand influences the relative reactivity of the two catalyst enantiomers, as shown by the comparison between catalysts Rh-1 and Rh-3. Taken together, these results are consistent with the known superior performance of Ir-based catalysts in the hydrogenation of sterically demanding, unfunctionalized olefins.

Importantly, however, complete hydrogenation alone does not imply stereochemical control. The decisive question is whether any of these catalysts can convert SQE into SQA with a non-statistical distribution of stereoisomers. This issue was addressed by quantitative ^13^C NMR spectroscopic analysis of the crude hydrogenation products, as discussed below.

### Quantitative ^13^C NMR spectroscopic assessment of stereochemical outcome

The stereochemical outcome of SQE hydrogenation was assessed by quantitative ^13^C NMR spectroscopy. While enantiomers are indistinguishable by ^13^C NMR spectroscopy, diastereomers give rise to distinct resonance patterns because nuclei located near stereogenic centres experience different local electronic environments.^42^ Thus, ^13^C NMR spectroscopy provides a direct method to evaluate the diastereomeric distribution of SQA without derivatization or chromatographic separation.

**Fig. 6:**
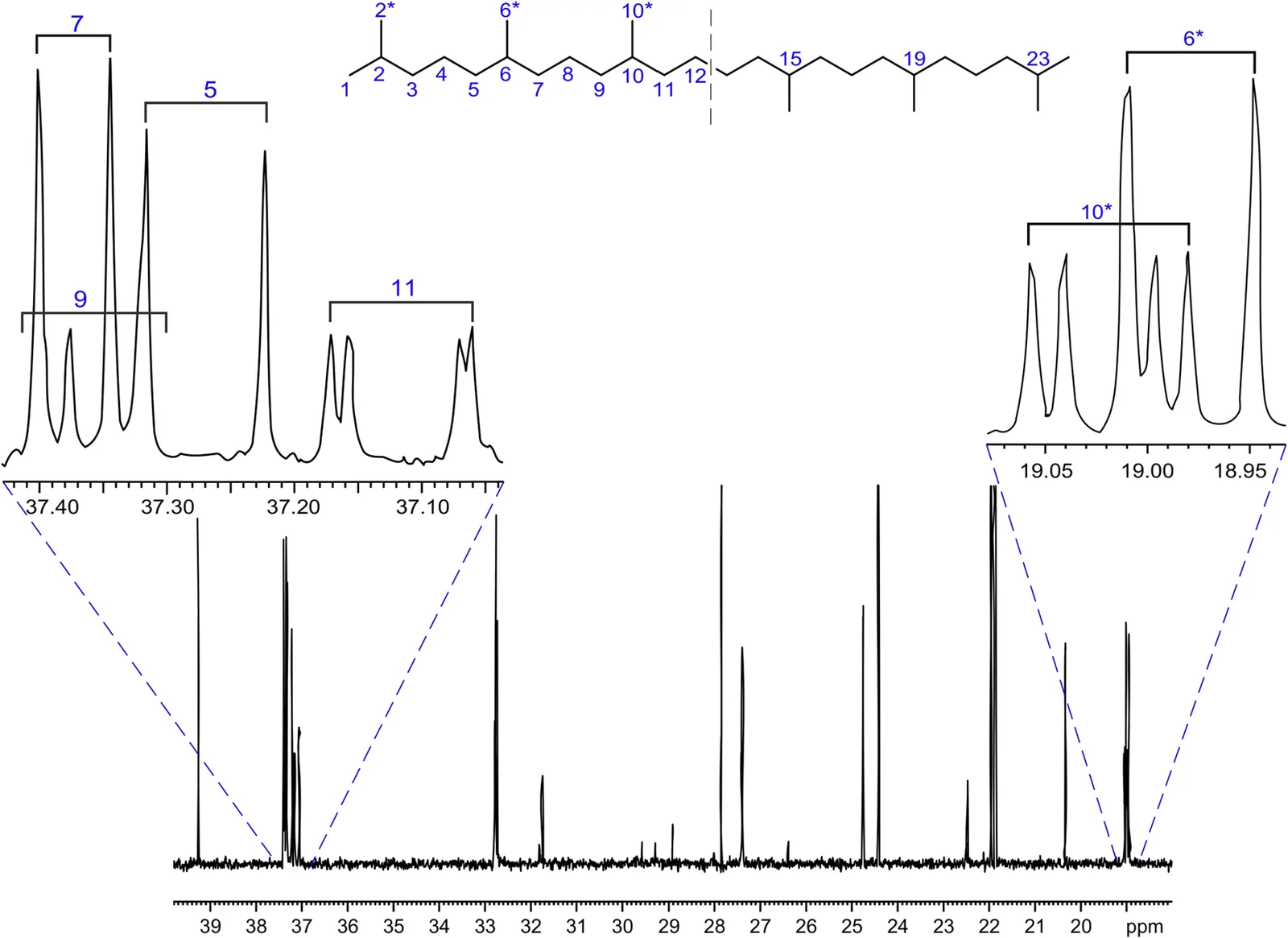
^13^C NMR spectrum of squalane from non-selective hydrogenation of squalene with Pd/C.

The ^13^C NMR spectrum of SQA, obtained by non-selective hydrogenation of SQE with Pd/C, was used as a reference for a statistical diastereomeric distribution (Fig. 6).

Chiral carbon atoms adjacent to achiral terminal groups (*e.g.*, C6) are influenced only by the chirality of the inner C atom (*e.g.*, C10).^42^ Accordingly, each resonance splits into two signals corresponding to two couples of (indistinguishable) enantiomers: *RR*/*SS* and *RS*/*SR*. In contrast, the internal chiral carbons (*e.g.*, C10) are influenced by the two chiral centres (*e.g.*, C6 and C15) and signals split into four peaks relative to the four couples of enantiomers: *RRR*/*SSS*, *RRS*/*SSR*, *RSS*/*SRR*, and *RSR*/*SRS*. Although complete assignment of all individual stereoisomeric resonances is not required, the resulting splitting pattern of signals is diagnostic of the diastereomeric population, and diastereomeric excess can be evaluated from peak integration. In this specific case of SQA, the resonances of methylene C11 are diagnostic for determining the diastereomeric excess, as they are well-resolved and free from overlap with other signals. The equal intensity of the two sets of C11 resonances in Fig. 6 indicates the absence of stereochemical bias, consistent with a statistical hydrogenation pathway. Nearly identical splitting patterns were observed for SQA samples obtained with Ir-1, Ir-2, and all Rh-based catalysts, indicating that these systems promote hydrogenation without measurable stereochemical induction under the employed conditions (see SI).

**Fig. 7:**
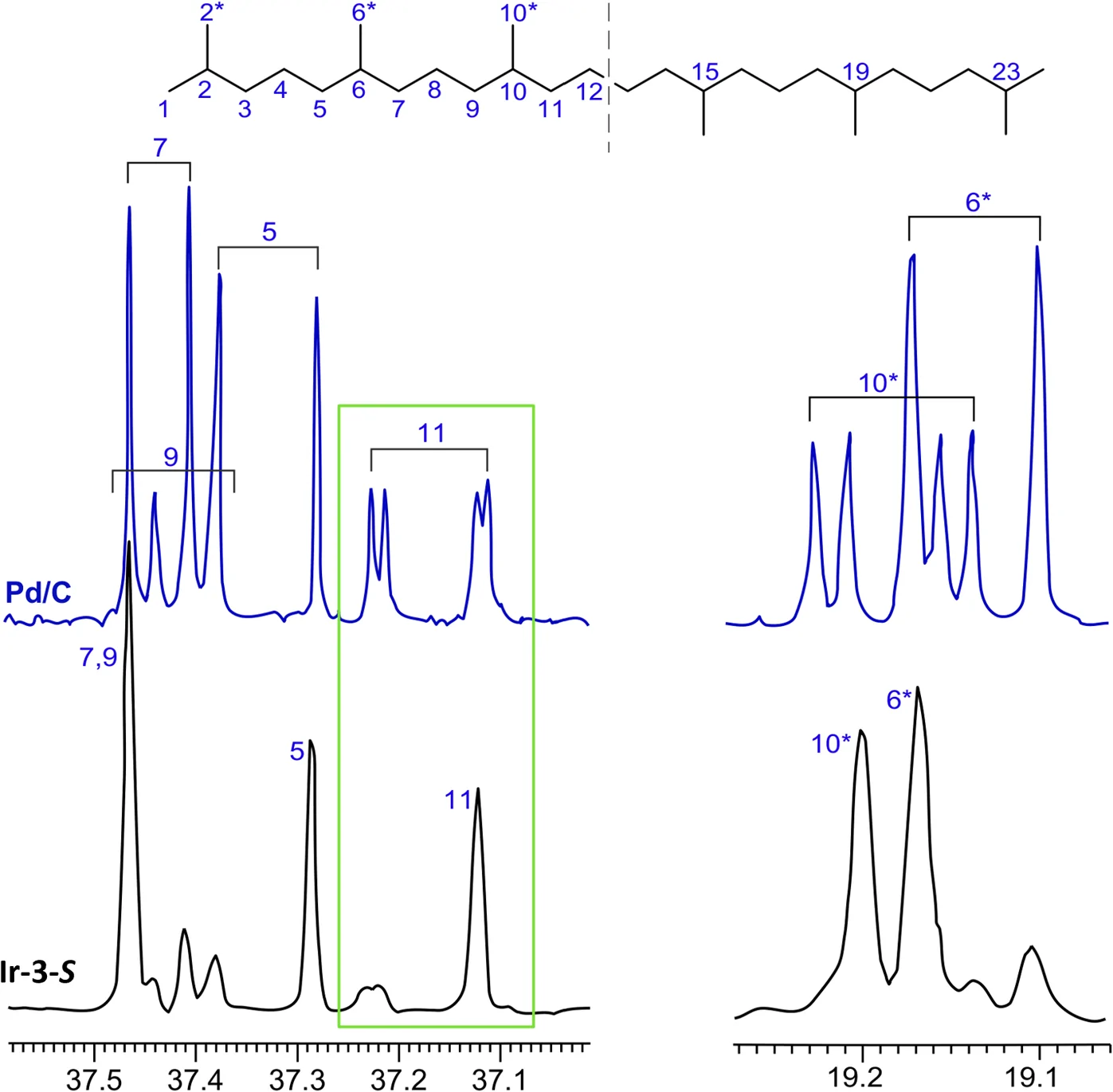
Expansions of the methylene and methyl regions of ^13^C NMR spectra of squalene hydrogenated with non-selective Pd/C (top) and selective Ir-3-*S* (bottom). Methylene C11 region used for %d.e. evaluation highlighted in green. Integration details and calculations are reported in Fig. S19 of the SI.

Remarkably, the ^13^C NMR spectrum of the SQA obtained with Ir-3-*S* displays a markedly simplified set of resonances; a clear fingerprint of an uneven diastereomeric distribution (Fig. 7, bottom).

Quantitative integration of the diagnostic C11 resonances revealed a strongly non-statistical product distribution, corresponding to a diastereomeric excess of *ca.* 90%. The integrated peak areas and the equation used to calculate the diastereomeric excess are provided in the SI (Fig. S19). Replicate experiments yielded comparable values within experimental error (±2%). Lower reaction temperatures could potentially enhance stereochemical discrimination and increase the d.e., although they may also reduce the hydrogenation rate and hinder complete conversion of the six double bonds. The influence of temperature and other reaction parameters on the stereochemical outcome will be investigated in future studies.

To the best of our knowledge, this represents the first catalytic access to stereoregular SQA. Such stereochemically biased saturated isoprenoid hydrocarbons may provide useful platforms for future studies of the relationship between stereochemical regularity and physicochemical or biological properties. This possibility is particularly relevant because squalane has been shown to partition into the midplane of synthetic lipid membranes and to affect membrane organization and permeability.^43^

### Qualitative comparison of catalyst steric environments

**Fig. 8:**
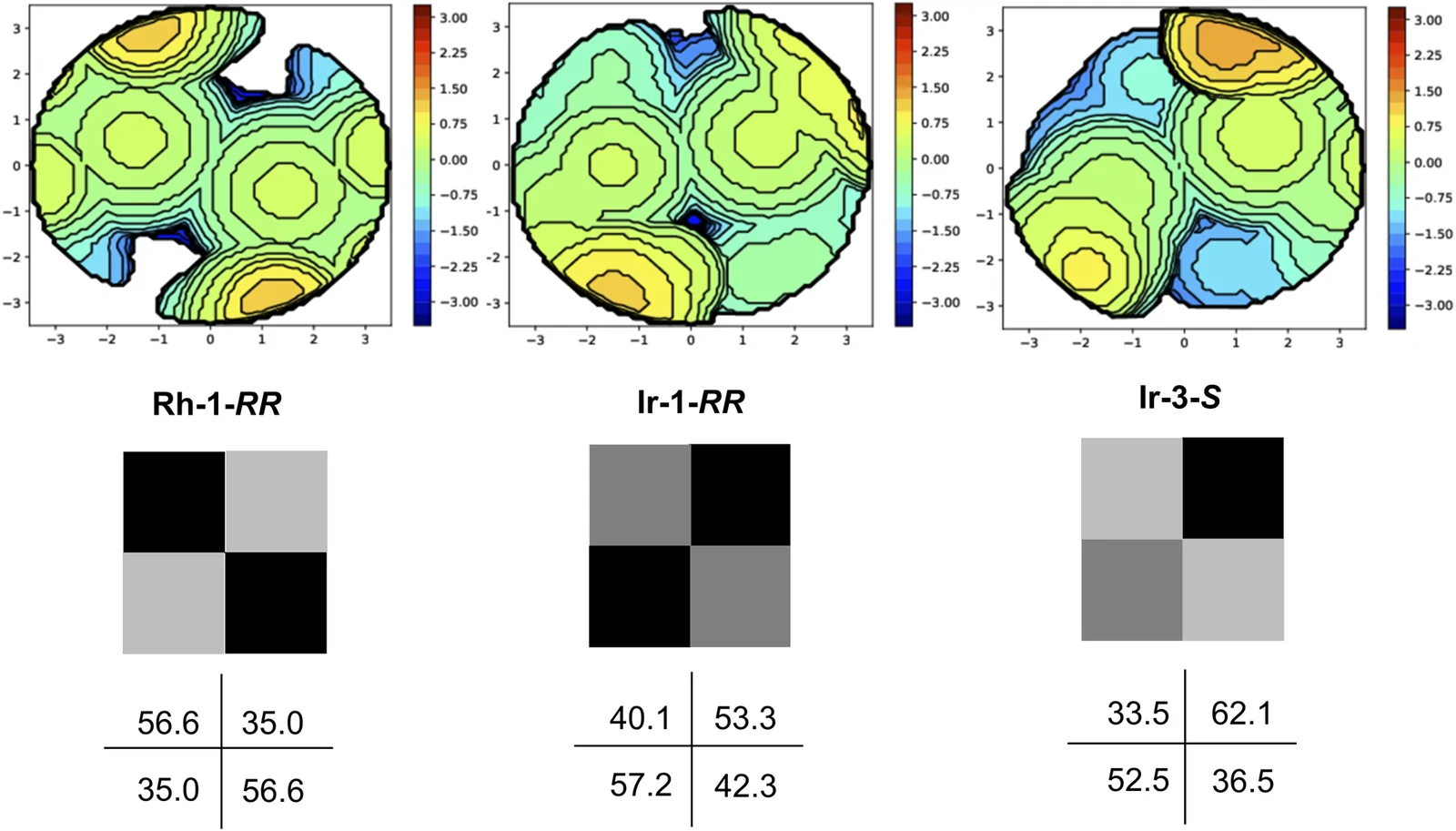
Comparison of the active pockets of three hydrogenation catalysts.

The behaviour of Ir-3-S is consistent with the known sensitivity of Ir–P,N hydrogenation catalysts to subtle differences in ligand steric environment, as previously observed by Pfaltz.^23^ To gain preliminary insight into the origin of the selectivity observed for Ir-3-*S*, the steric environments of Ir-3-*S* and of two representative non-selective catalysts, Ir-1-*RR* and Rh-1-*RR*, were compared using Cavallo's buried-volume analysis^44,45^ as implemented in SambVca 2.0 (Fig. 8). The two non-selective reference catalysts, Rh-1-*RR* and Ir-1-*RR*, display comparatively less discriminating steric environments. Rh-1-*RR* exhibits a *C*_2_*-*symmetric active pocket with two relatively open (%*V*_Bur_ = 35.5) and two occupied quadrants (%*V*_Bur_ = 56.6). Although Ir-1-*RR* formally shows *C*_1_ symmetry, the differences between equivalent quadrants remain small, with %*V*_bur_ values of 40.1 and 42.3 for the less occupied quadrants and 53.3 and 57.2 for the more occupied quadrants, indicating only a modest deviation from *C*_2_-symmetry. In contrast, Ir-3-*S* displays a more pronounced *C*_1_-symmetry, with the two empty quadrants being similarly occupied (%*V*_Bur_ = 33.5 and 36.5, respectively) and the two occupied quadrants differing significantly (%*V*_Bur_ = 52.5 and 62.1, respectively). Thus, Ir-3-*S* combines a relatively accessible coordination pocket with a markedly asymmetric steric profile. Such a steric profile is consistent with preferential substrate orientations during hydrogenation, although a detailed mechanistic assignment is beyond the scope of the present study.

Steric discrimination between methyl and longer alkyl substituents is one of the most difficult selectivity problems in asymmetric hydrogenation of unfunctionalized olefins. The steric-map analysis is consistent with the view that stereochemical induction in SQE hydrogenation benefits from both accessibility of the active pocket and an asymmetric ligand environment. This analysis should be regarded as a qualitative structural rationale rather than a complete mechanistic explanation; nevertheless, it provides a useful starting point for discussing the different stereochemical outcomes observed within the catalyst set.

## Conclusion

In this work, we demonstrated that a stereochemically biased squalane distribution can be obtained through the complete hydrogenation of a flexible, unfunctionalized hydrocarbon polyene. Using squalene as a demanding model substrate, a focused library of Ir- and Rh-based homogeneous hydrogenation catalysts was evaluated under standardized conditions and benchmarked against Pd/C.

All Ir-based complexes achieved complete hydrogenation to squalane under the employed conditions, whereas the Rh-based catalysts showed lower and more variable activity. However, complete conversion did not necessarily translate into stereochemical control. Among all investigated systems, Ir-3-*S* combined full hydrogenation with pronounced stereochemical induction, affording squalane with a highly non-statistical diastereomeric distribution corresponding to *ca.* 90% diastereomeric excess. To the best of our knowledge, this represents the first catalytic access to stereoregular squalane.

A key enabling feature of the study was the direct evaluation of both hydrogenation efficiency and stereochemical outcome from crude reaction mixtures. Parallel miniaturized hydrogenation experiments, combined with quantitative ^1^H and ^13^C NMR spectroscopic analysis, allowed rapid comparison of catalyst performance without product isolation, derivatization, or chromatographic separation. Diagnostic ^13^C NMR resonances provided a direct means of distinguishing statistical from stereochemical-biased squalane distributions.

Buried-volume analysis using SambVca provided a qualitative comparison between the selective Ir-3-S catalyst and representative non-selective systems. Compared with representative non-selective catalysts, Ir-3-*S* combines an accessible coordination region with a markedly asymmetric steric profile, a feature consistent with preferential substrate orientation during hydrogenation, although a detailed mechanistic assignment remains beyond the scope of the present study.

These findings show that complete catalytic hydrogenation of squalene can provide access to stereochemically biased squalane distributions when an appropriate Ir–P,N catalyst is used. More broadly, the combination of miniaturized catalyst screening and direct NMR-based stereochemical analysis provides a practical approach to evaluate stereochemical bias in the hydrogenation of complex isoprenoid hydrocarbons. Access to stereoregular squalane also opens opportunities to investigate whether stereochemical regularity affects the physicochemical or biological properties of fully saturated isoprenoid hydrocarbons, in line with the broader influence of stereochemical order on material properties and function.^46^

## Experimental section

### General information

Hydrogenation experiments were performed using a Freeslate, formerly Symyx, Parallel Pressure Reactor, PPR48, platform consisting of 48 reaction cells arranged in six 8-cell modules. The PPR setup is fully integrated into a triple MBraun LabMaster glovebox operated under dry nitrogen and has been described elsewhere.^35–41^

### Hydrogenation protocol

Before loading the reaction cells, the modules undergo ‘Bake&Purge’ cycles overnight (*i.e.* an intermittent flow of dry N_2_ at 90–140 °C over 8 h) to remove any impurity left from previous experiments. After cooling the modules to glovebox temperature, the stir tops are removed to fit the 48 cells with disposable glass vials and titanium stir paddles and then set back in place. Next, an N_2_ pressure test is performed to make sure that all reactors and fittings are tight at the operating pressure. The glass vials are loaded with the 4.5 mL of 1,2-difluorobenzene (*o*DFB) and 17 mg of SQE prior to closing the modules. Subsequently, the modules are thermostated at 50 °C, stirring is started, the cells are pressurized with hydrogen at *p*(H_2_) = 200 psi, and the catalyst injection sequence is started. The catalyst solutions are uploaded into the needle and subsequently injected into the cell of destination (0.5 mL), thus starting the reaction. The process is allowed to proceed under stirring at constant temperature and pressure and then quenched by venting the reactors after 2 h. When all cells have been quenched, the modules are cooled to glovebox temperature, the stir tops are removed, and the vials containing the reaction phase are recovered and sent for product characterization.

### NMR characterization

0.5 mL of the reaction phase is transferred into an NMR tube containing 0.1 mL of C_6_D_6_. NMR spectra were recorded with a Bruker Avance III 400 spectrometer (400 MHz for ^1^H) equipped with a 5 mm Prodigy high-temperature cryoprobe and an autosampler with 24 positions. Operating conditions were as follows: ^1^H NMR: 90° pulse width; 32 K time domain data points; 8.0 kHz spectral width; 2 s acquisition time; 10 s relaxation delay; 1–8 transients. ^13^C NMR: 45° pulse; acquisition time, 2.7 s; relaxation delay, 5.0 s; 2 K transients. Broad band proton decoupling was achieved with a modified WALTZ16 sequence (BI_WALTZ16_32 by Bruker).

### Computational details

The CREST software was used to sample the conformer space of the catalysts, and the lowest conformer was further optimized at the TPSSh/cc-pVDZ level. SambVCa 2.0 was used to analyse and visualize the active pockets.^47,48^ To place the coordinate system, the metal was used to define the center, the four sp^2^-hybridized carbons of the cyclooctadiene (COD) were used to define the *z*-axis, while the two sp^2^-hybridized carbons of COD on the side of the phosphine ligand were used to define the direction of the *y*-axis.

## Conflicts of interest

A provisional patent application covering aspects of the stereoselective hydrogenation of squalene described in this manuscript has been filed by the University of Florida (Application No. 63/996 536). The authors declare no other competing interests.

## Supplementary Material

RA-OLF-D6RA05192H-s001

RA-OLF-D6RA05192H-s002

## Data Availability

The data supporting this article have been included as part of the supplementary information (SI). Supplementary information: detailed experimental data and NMR characterization. See DOI: https://doi.org/10.1039/d6ra05192h.

## References

[cit1] Jacobsen E. N. , Pfaltz A. and Yamamoto H., Comprehensive Asymmetric Catalysis I-III with Contributions by Numerous Experts, Springer-Verlag, Berlin, 1999

[cit2] Noyori R. (2002). Asymmetric Catalysis: Science and Opportunities. Angew. Chem., Int. Ed..

[cit3] Knowles W. S. (1983). Asymmetric Hydrogenation. Acc. Chem. Res..

[cit4] Seo C. S. G., Morris R. H. (2019). Catalytic Homogeneous Asymmetric Hydrogenation: Successes and Opportunities. Organometallics.

[cit5] Shultz C. S., Krska S. W. (2007). Unlocking the Potential of Asymmetric Hydrogenation at Merck. Acc. Chem. Res..

[cit6] Genet J. P. (2003). Asymmetric Catalytic Hydrogenation. Design of New Ru Catalysts and Chiral Ligands: From Laboratory to Industrial Applications. Acc. Chem. Res..

[cit7] Blacker A. J. and Thompson P., in Asymmetric Catalysis on Industrial Scale: Challenges, Approaches and Solutions, ed. H.-U. Blaser and H.-J. Federsel, Wiley-VCH, Weinheim, 2^nd^ edn, 2010, vol. 16, pp. 265–290

[cit8] Noyori R. (2013). Facts are the Enemy of Truth-reflections on Serendipitous Discovery and Unforeseen Developments in Asymmetric Catalysis. Angew. Chem., Int. Ed..

[cit9] Ager D. J., De Vries A. H. M., de Vries J. G. (2012). Asymmetric Homogeneous Hydrogenations at Scale. Chem. Soc. Rev..

[cit10] Hayler J. D., Leahy D. K., Simmons E. M. (2018). A Pharmaceutical Industry Perspective on Sustainable Metal Catalysis. Organometallics.

[cit11] Busacca C. A., Fandrick D. R., Song J. J., Senanayake C. H. (2011). The Growing Impact of Catalysis in the Pharmaceutical Industry. Adv. Synth. Catal..

[cit12] Blaser H.-U. (2002). The Chiral Switch of (S)-Metolachlor: A Personal Account of an Industrial Odyssey in Asymmetric Catalysis. Adv. Synth. Catal..

[cit13] Soeherman J. K., Kim J., Onn T. M., Reineke T., Dauenhauer P. J. (2024). Catalytic Hydrogenation of Alkylphenols for Renewable Caprolactone. ACS Sustain. Chem. Eng..

[cit14] Xu Y., Liang Y., Guo H., Qi X. (2023). Catalytic hydrogenation of levulinic acid to γ-valerolactone over lignin-metal coordinated carbon nanospheres in water. Int. J. Biol. Macromol..

[cit15] Trost B. M. (2002). On Inventing Reactions for Atom Economy. Acc. Chem. Res..

[cit16] Margarita C., Andersson P. G. (2017). Evolution and Prospects of the Asymmetric Hydrogenation of Unfunctionalized Olefins. J. Am. Chem. Soc..

[cit17] Wang A., Fraga R. P. A., Hörmann E., Pfaltz A. (2011). Iridium-Catalyzed Asymmetric Hydrogenation of Unfunctionalized, Trialkyl-substituted Olefins. Chem.–Asian J..

[cit18] Diéguez M. and Pizzano A., Metal-catalyzed Asymmetric Hydrogenation: Evolution and Prospect, Academic Press, Cambridge MA, 2021

[cit19] Bell S., Wüstenberg B., Kaiser S., Menges F., Netscher T., Pfaltz A. (2006). Asymmetric Hydrogenation of Unfunctionalized, Purely Alkyl-Substituted Olefins. Science.

[cit20] Carroll M. P., Guiry P. J. (2014). P,N Ligands in Asymmetric Catalysis. Chem. Soc. Rev..

[cit21] Margalef J. , Pàmies O. and Diéguez M., in Iridium Catalysts for Organic Reactions, ed. L. A. Oro and C. Claver, Springer, Cham, 1^st^ edn, 2021, vol. 5, pp. 153–206

[cit22] Roseblade S. J., Pfaltz A. (2007). Recent Advances in Iridium-Catalysed Asymmetric Hydrogenation of Unfunctionalised Olefins. C.R. Chimie.

[cit23] Schrems M. G., Neumann E., Pfaltz A. (2007). Indium-catalyzed Asymmetric Hydrogenation of Unfunctionalized Tetrasubstituted Olefins. Angew. Chem., Int. Ed..

[cit24] Crispino G. A., Ho P. T., B Sharpless K. (1993). Selective Perhydroxylation of Squalene: Taming the Arithmetic Demon. Science.

[cit25] Cui X., Ogle J. W., Burgess K. (2005). Stereoselective Hydrogenations of Aryl-substituted Dienes. Chem. Commun..

[cit26] Cui X., Burgess K. (2005). Catalytic Homogeneous Asymmetric Hydrogenations of Largely Unfunctionalized Alkenes. Chem. Rev..

[cit27] Muramatsu H., Kawano H., Ishii Y., Saburi M., Uchida Y. (1989). Double Asymmetric Hydrogenation of Conjugated Dienes Catalysed by Ruthenium Binap Complexes. J. Chem. Soc. Chem. Commun..

[cit28] Loscher S., Schobert R. (2013). Total Synthesis and Absolute Configuration of Epicoccamide D, a Naturally Occurring Mannosylated 3-Acyltetramic Acid. Chem. – Eur. J..

[cit29] Morgan N. R., Magalingam K. B., Radhakrishnan A. K., Arumugam M., Jamil A., Bhuvanendran S. (2024). Explicating the Multifunctional Roles of Tocotrienol and Squalene in Promoting Skin Health. Skin Health Dis..

[cit30] Sánchez-Quesada C., López-Biedma A., Toledo E., Gaforio J. J. (2018). Squalene Stimulates a Key Innate Immune Cell to Foster Wound Healing and Tissue Repair. J. Evidence-Based Complementary Altern. Med..

[cit31] Wolosik K., Chalecka M., Gasiewska G., Palka J., Surazynski A. (2025). Squalane as a Promising Agent Protecting UV-Induced Inhibition of Collagen Biosynthesis and Wound Healing in Human Dermal Fibroblast. Molecules.

[cit32] Ciriminna R., Pandarus V., Beland F., Pagliaro M. (2014). Catalytic Hydrogenation of Squalene to Squalane. Org. Process Res. Dev..

[cit33] Kim S., Karadeniz F. (2012). Biological importance and applications of squalene and squalane. Adv. Food Nutr. Res..

[cit34] Pandarus V., Ciriminna R., Béland F., Pagliaro M., Kaliaguine S. (2017). Solvent-Free Chemoselective Hydrogenation of Squalene to Squalane. ACS Omega.

[cit35] Ehm C., Mingione A., Vittoria A., Zaccaria F., Cipullo R., Busico V. (2020). High-Throughput Experimentation in Olefin Polymerization Catalysis: Facing the Challenges of Miniaturization. Ind. Eng. Chem. Res..

[cit36] Ehm C., Vittoria A., Goryunov G. P., Kulyabin P. S., Budzelaar P. H. M., Voskoboynikov A. Z., Busico V., V Uborsky D., Cipullo R. (2018). Connection of Stereoselectivity, Regioselectivity, and Molecular Weight Capability in *rac*-R′_2_Si(2-Me-4-R-indenyl)_2_ZrCl_2_ Type Catalysts. Macromolecules.

[cit37] V Uborsky D., Mladentsev D. Y., Guzeev B. A., Borisov I. S., Vittoria A., Ehm C., Cipullo R., Hendriksen C., Friederichs N., Busico V., Voskoboynikov A. Z. (2020). C 1-Symmetric Si-bridged (2-indenyl)(1-indenyl) Ansa-metallocenes as Efficient Ethene/1-hexene Copolymerization Catalysts. Dalton Trans..

[cit38] Kulyabin P. S., Goryunov G. P., Sharikov M. I., V Izmer V., Vittoria A., Budzelaar P. H. M., Busico V., Voskoboynikov A. Z., Ehm C., Cipullo R., V Uborsky D. (2021). ansa-Zirconocene Catalysts for Isotactic-Selective Propene Polymerization at High Temperature: A Long Story Finds a Happy Ending. J. Am. Chem. Soc..

[cit39] Ehm C., Vittoria A., Goryunov G. P., V Izmer V., Kononovich D. S., V Samsonov O., Budzelaar P. H. M., Voskoboynikov A. Z., Busico V., V Uborsky D., Cipullo R. (2020). On the Limits of Tuning Comonomer Affinity of ‘Spaleck-type’ ansa-zirconocenes in Ethene/1-hexene Copolymerization: a High-Throughput Experimentation/QSAR Approach. Dalton Trans..

[cit40] Ehm C., Vittoria A., Goryunov P. G., Izmer V. V., Kononovich S. D., Samsonov V. O., Di Girolamo R., Budzelaar H. M. P., Voskoboynikov Z. A., Busico V., Uborsky V. D., Cipullo R. (2020). An Integrated High Throughput Experimentation/Predictive QSAR Modeling Approach to *ansa*-Zirconocene Catalysts for Isotactic Polypropylene. Polymers.

[cit41] Cannavacciuolo F. D., Yadav R., Esper A., Vittoria A., Antinucci G., Zaccaria F., Cipullo R., Budzelaar P. H. M., Busico V., Goryunov G. P., V Uborsky D., Voskoboynikov A. Z., Searles K., Ehm C., Veige A. S. (2022). A High-Throughput Approach to Repurposing Olefin Polymerization Catalysts for Polymer Upcycling. Angew. Chem., Int. Ed..

[cit42] Dalling D. K., Pugmire R. J., Grant D. M., Hull W. E. (1986). The Use of High-Field Carbon-13 NMR Spectroscopy to Characterize Chiral Centers in Isopranes. Magn. Reson. Chem..

[cit43] Salvador-Castell M., Golub M., Erwin N., Brooks N. J., Winter R., Peters J., Oger P. M. (2021). Characterisation of a synthetic Archeal membrane reveals a possible new adaptation route to extreme conditions. Commun. Biol..

[cit44] Falivene L., Cavallo L., Talarico G. (2015). Buried Volume Analysis for Propene Polymerization Catalysis Promoted by Group 4 Metals: A Tool for Molecular Mass Prediction. ACS Catal..

[cit45] Poater A., Cavallo L. (2009). Comparing Families of Olefin Polymerization Precatalysts using the Percentage of Buried Volume. Dalton Trans..

[cit46] Worch J., Prydderch H., Jimaja S., Bexis P., Becker M. L., Dove A. (2019). Stereochemical enhancement of polymer properties. Nat. Rev. Chem..

[cit47] Falivene L., Cao Z., Petta A., Serra L., Poater A., Oliva R., Scarano V., Cavallo L. (2019). Towards the Online Computer-Aided Design of Catalytic Pockets. Nat. Chem..

[cit48] Falivene L., Credendino R., Poater A., Petta A., Serra L., Oliva R., Scarano V., Cavallo L. (2016). SambVca 2. A Web Tool for Analyzing Catalytic Pockets with Topographic Steric Maps. Organometallics.

