# Supplementary material for: Ir/Rh-catalyzed hydrogenation of squalene: access to stereoregular squalane
Source: RSC Adv. 2026 Sep 7;16(47):61750–6. doi: 10.1039/d6ra05192h (PMC13548352; doi:10.1039/d6ra05192h)
Supplement: Supplementary file 1 [file RA-016-D6RA05192H-s001.pdf]

Supplementary Information (SI) for RSC Advances.
This journal is © The Royal Society of Chemistry 2026

Ir/Rh-Catalyzed Hydrogenation of Squalene: Access to Stereoregular Squalane

Teresa Iovine[a,c], Christian Ehm*[a,c], Umesh I. Kaluarachchige Don[b], Vincenzo Busico[a,c], Adam S. Veige*[b], Roberta Cipullo*[a,c]

[a] *Dipartimento di Scienze Chimiche, Università di Napoli Federico II, 80126 Napoli, Italy.*

[b] *Department of Chemistry, University of Florida, Center for Catalysis, P.O. Box 117200, Gainesville, Florida 32611, United States.*

[c] *DPI, P.O. Box 902, 5600 AX Eindhoven, the Netherlands.*

Supporting Information

1. NMR Characterization of Squalene 1
2. NMR Characterization of Squalane obtained by Squalene Hydrogenation 4
3. ^{13}C NMR spectra of Hydrogenated Squalene from All Catalysts 5
4. Hydrogenation Experiment Data 10

1. NMR Characterization of Squalene

Completely assigned ^{1}H and ^{13}C NMR spectra of squalene are reported in **Figure S1** and **Figure S2**, respectively. 2D NMR experiments allowed to assign all resonances, as explained below.

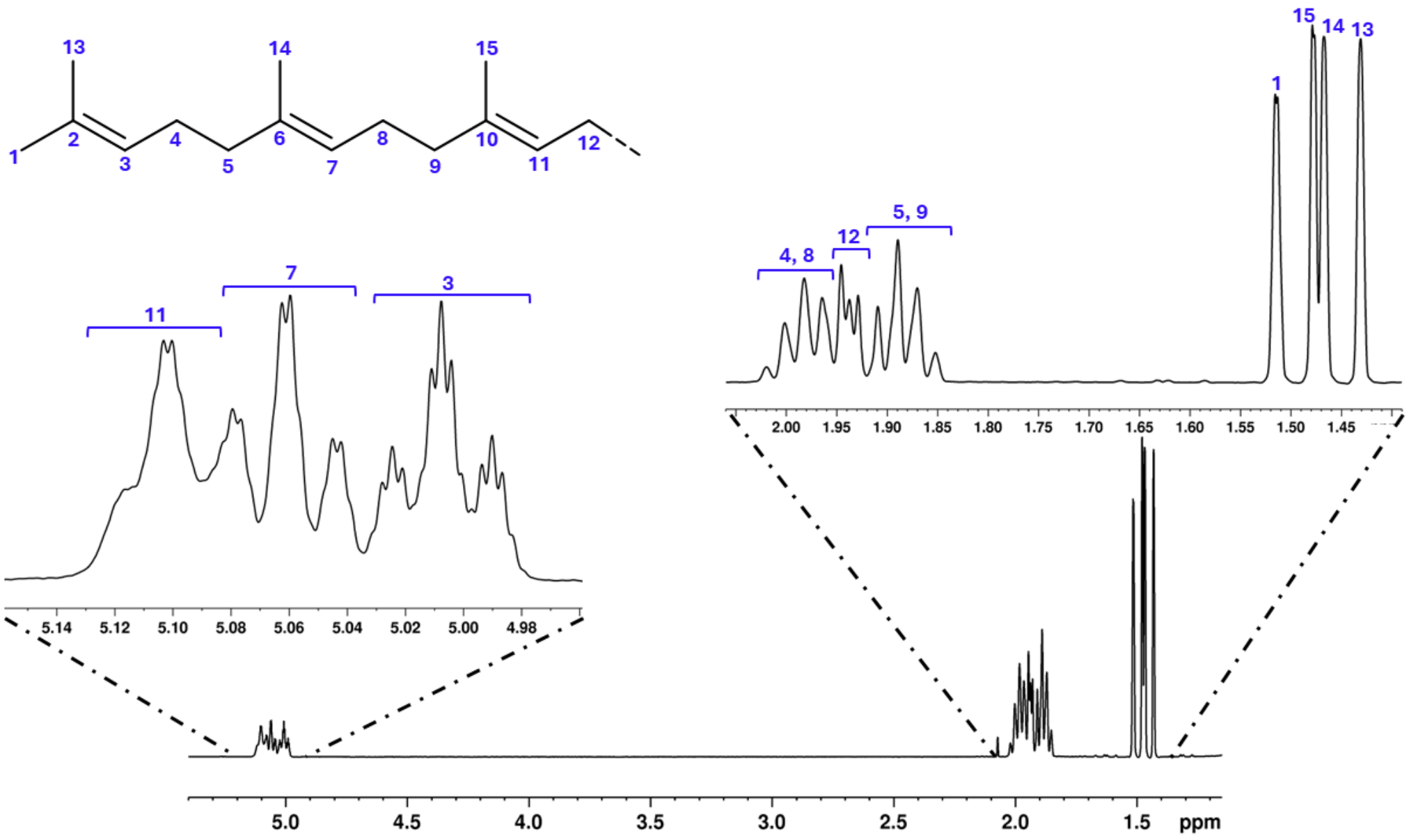

Figure S1. ^{1}H NMR spectrum of squalene in *o*DFB.

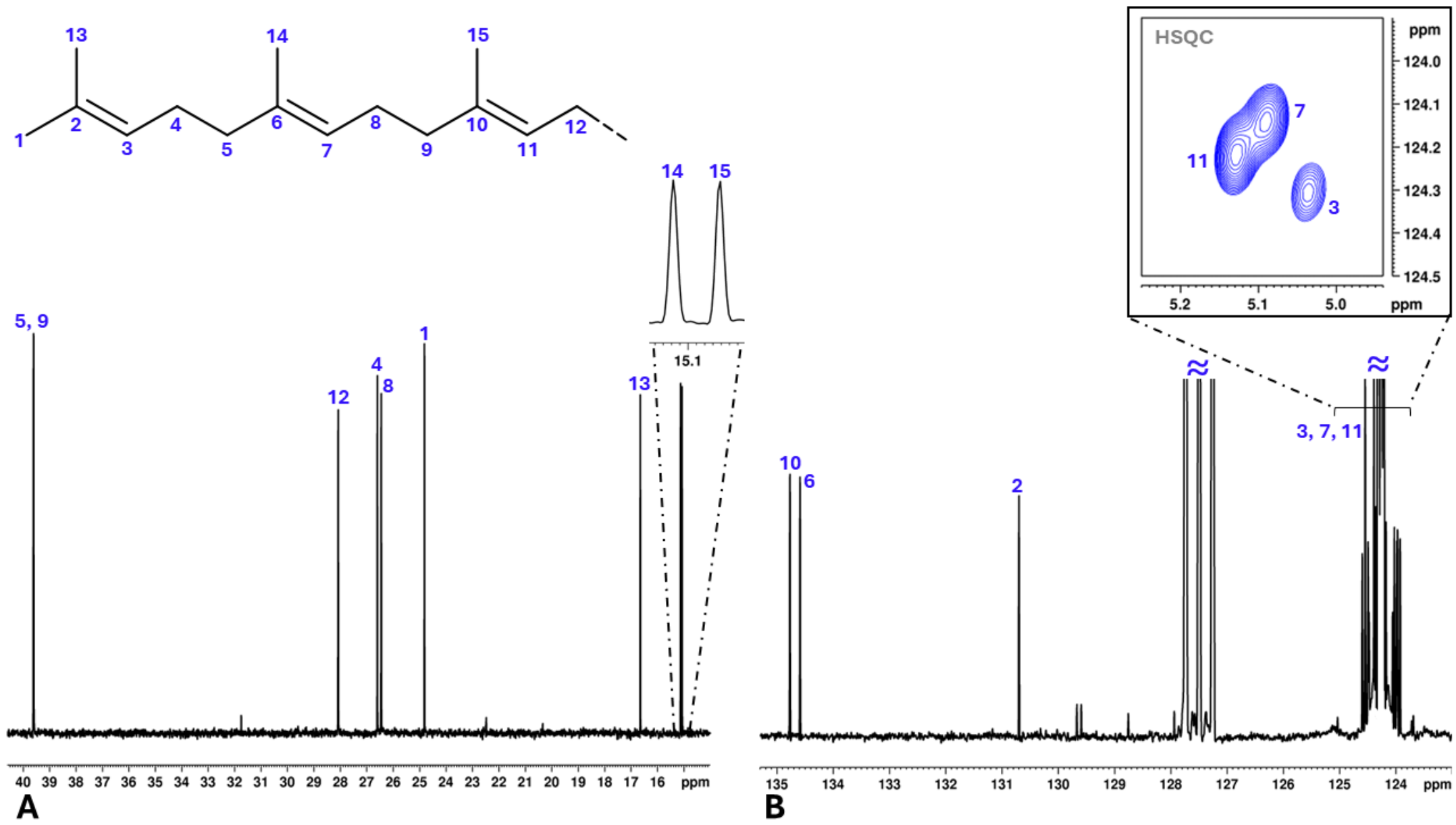

Figure S2. ^{13}C NMR spectrum of squalene in *o*DFB.

The HSQC experiment (**Figure S3**) showed scalar coupling between peaks resonating at $\delta(^1H)$ = 1.46, 1.50, 1.51, 1.55 ppm and resonances at $\delta(^{13}C)$ = 16.6, 14.9, 15.0, 24.7 ppm, respectively, attributable to the four methyl groups of squalene (**1**, **13**, **14**, **15**).

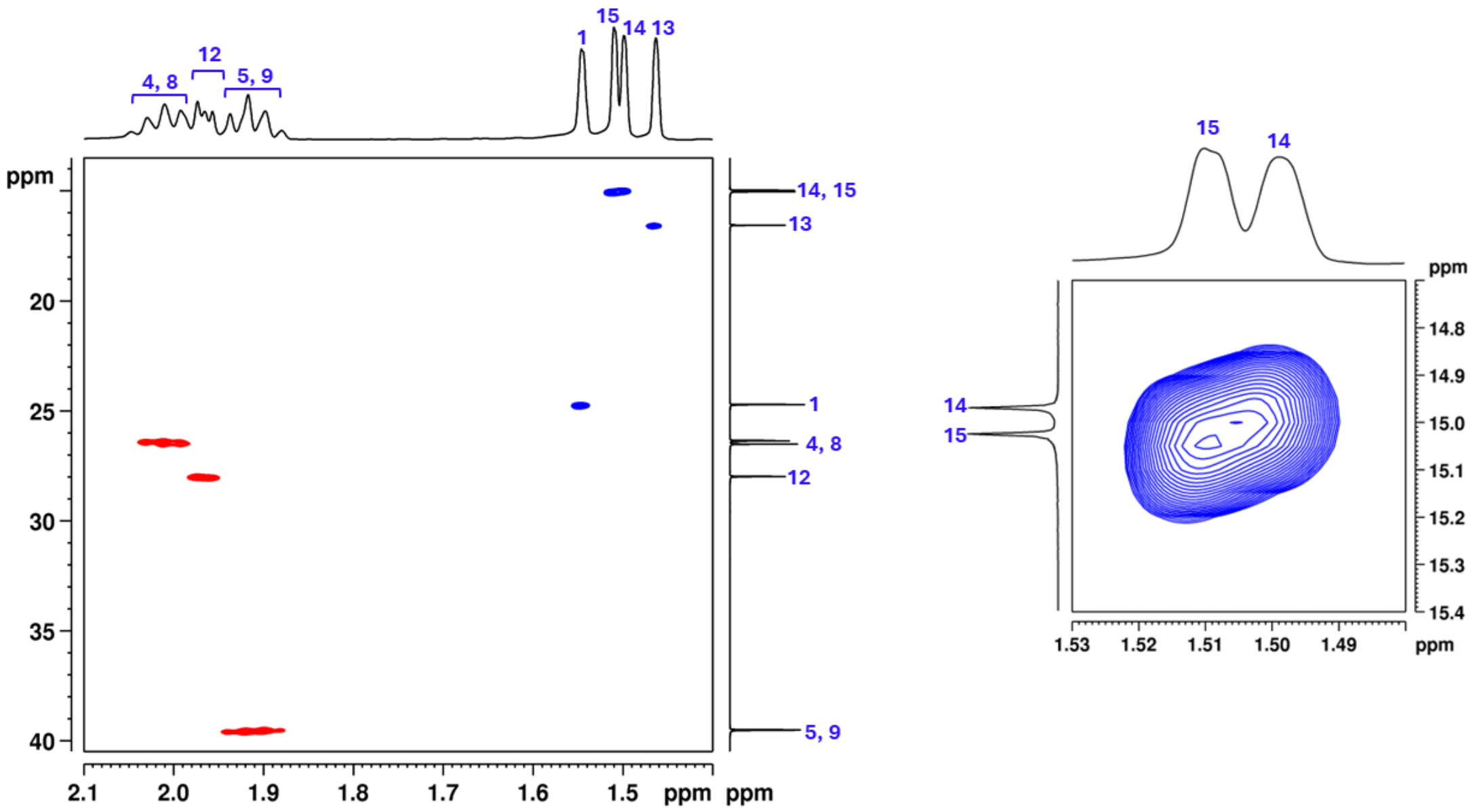

Figure S3. Expansion of the aliphatic region (left) and the methyl region (right) of the HSQC spectrum of squalene.

From the HMBC experiment (**Figure S4**), we were able to identify the two terminal methyl groups (**1**, **13**), since there are long-range scalar correlations between the resonance at $\delta(^1H)$ = 1.46 ppm (1.55 ppm) and $\delta(^{13}C)$ = 24.7 ppm (16.6 ppm), and the quaternary carbon atom to which they are bonded, resonating at $\delta(^{13}C)$ = 130.6 ppm (**2**). Similarly, the two internal methyl groups at $\delta(^1H)$ = 1.50 and 1.51 ppm are bonded to the quaternary carbon atoms at $\delta(^{13}C)$ = 134.5 and 134.7 ppm (**6**, **10**) respectively (confirmation for the resonances of the quaternary carbon atoms comes from the HSQC experiment, which shows no correlation with any proton signal). From the HMBC experiment, it is also possible to identify the olefinic carbon atoms adjacent to the quaternary ones since there are correlation between the methyl protons at $\delta(^1H)$ = 1.46 and 1.55 ppm with the carbon atom resonating at $\delta(^{13}C)$ = 124.3 ppm (**3**), while the other two methyl groups at $\delta(^1H)$ = 1.50 and 1.51 ppm correlate with $\delta(^{13}C)$ = 124.1 and 124.2 ppm (**7**, **11**), respectively.

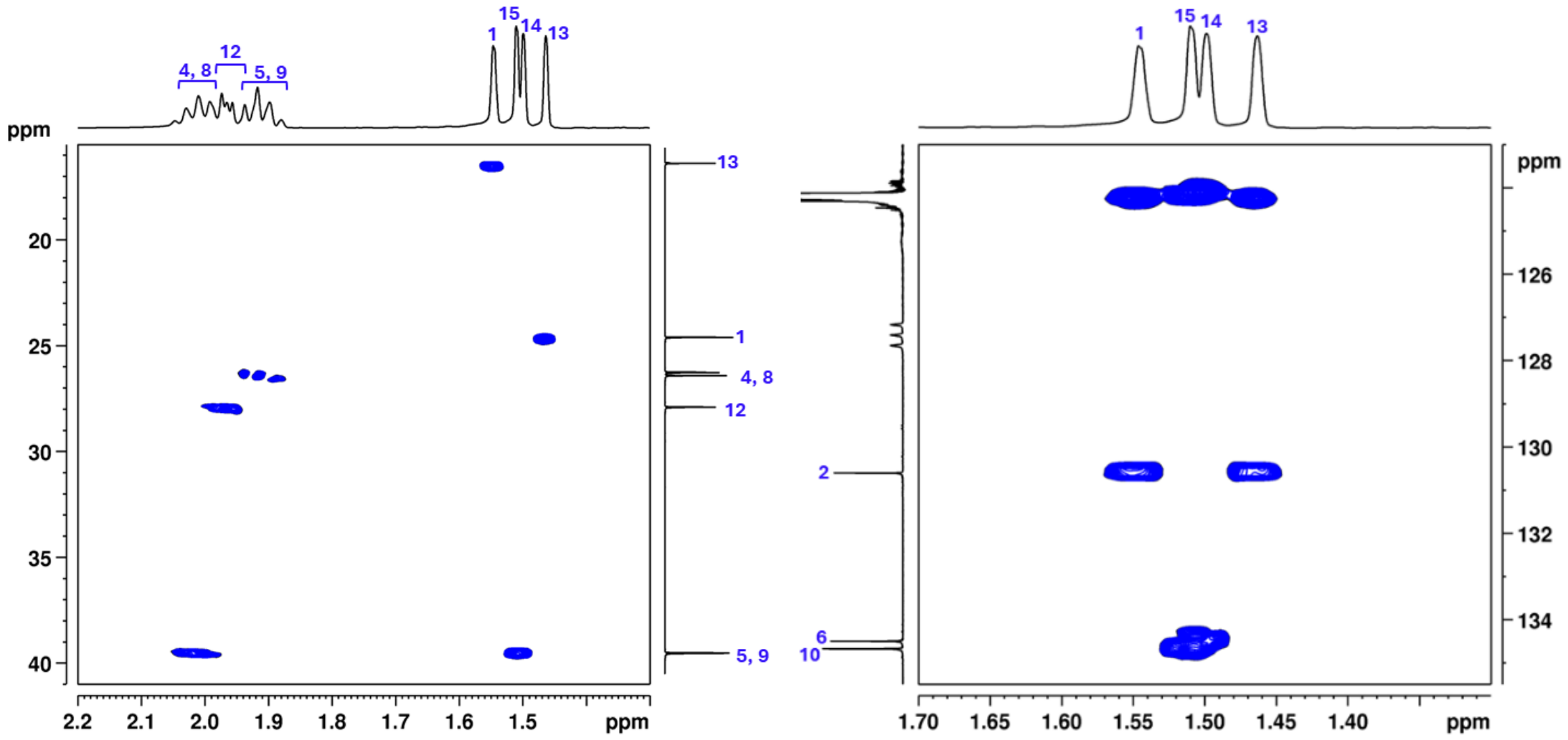

Figure S4. Expansions of the HMBC spectrum of squalene.

The resonances of the olefinic carbon atoms **3**, **7** and **11** were hidden by the *o*DFB signals and were recognized with the help of the HSQC spectrum, as reported in the spectrum in **Figure S2**, indicating scalar correlation between the methine protons at $\delta(^{1}\text{H})$ = 5.03, 5.09 and 5.13 ppm and the carbon atoms resonating at $\delta(^{13}\text{C})$ = 124.3, 124.1 and 124.2 ppm. Correlation between the signals at $\delta(^{1}\text{H})$ = 5.03 ppm and $\delta(^{13}\text{C})$ = 26.5 ppm in the HMBC spectrum (**Figure S5**) and correlation of the latter with $\delta(^{1}\text{H})$ = 2.01 ppm in the HSQC spectrum (**Figure S3**) allowed us to assign methylene **4**. In the same way, $\delta(^{1}\text{H})$ = 2.01 ppm correlating with $\delta(^{13}\text{C})$ = 39.5 ppm and the latter correlating with methyl groups **14** and **15** (**Figure S4**) allowed us to assign **5** and **9**, considering also peak integration (x2). The HMBC spectrum (**Figure S5**) shows also a correlation between $\delta(^{1}\text{H})$ = 5.09 ppm, which was assigned to **7**, and $\delta(^{13}\text{C})$ = 26.4 ppm, allowing the identification of **8**. The assignment of **7** is confirmed by the correlation with the resonances of the internal methyl groups and the methylenes at 39.5 ppm. The last-remaining olefinic methine **11** is then assigned to the resonances $\delta(^{1}\text{H})$ = 5.13 ppm and $\delta(^{13}\text{C})$ = 124.2 ppm, which led also to the identification of **12** at $\delta(^{13}\text{C})$ = 28.0 ppm, which **11** correlates to. This assignment is confirmed by the ROESY experiment (**Figure S5**), which show spatial correlation between the olefin protons at $\delta(^{1}\text{H})$ = 5.09 and 5.13 ppm and the methyl protons at $\delta(^{1}\text{H})$ = 1.50 and 1.51 ppm, respectively, clarifying the assignment of **14** and **15**. It is worth noting that the assignment of the resonances of the *cis*- and *trans*- terminal methyl groups (**1** and **13** respectively) agrees with prevision of chemical shifts.

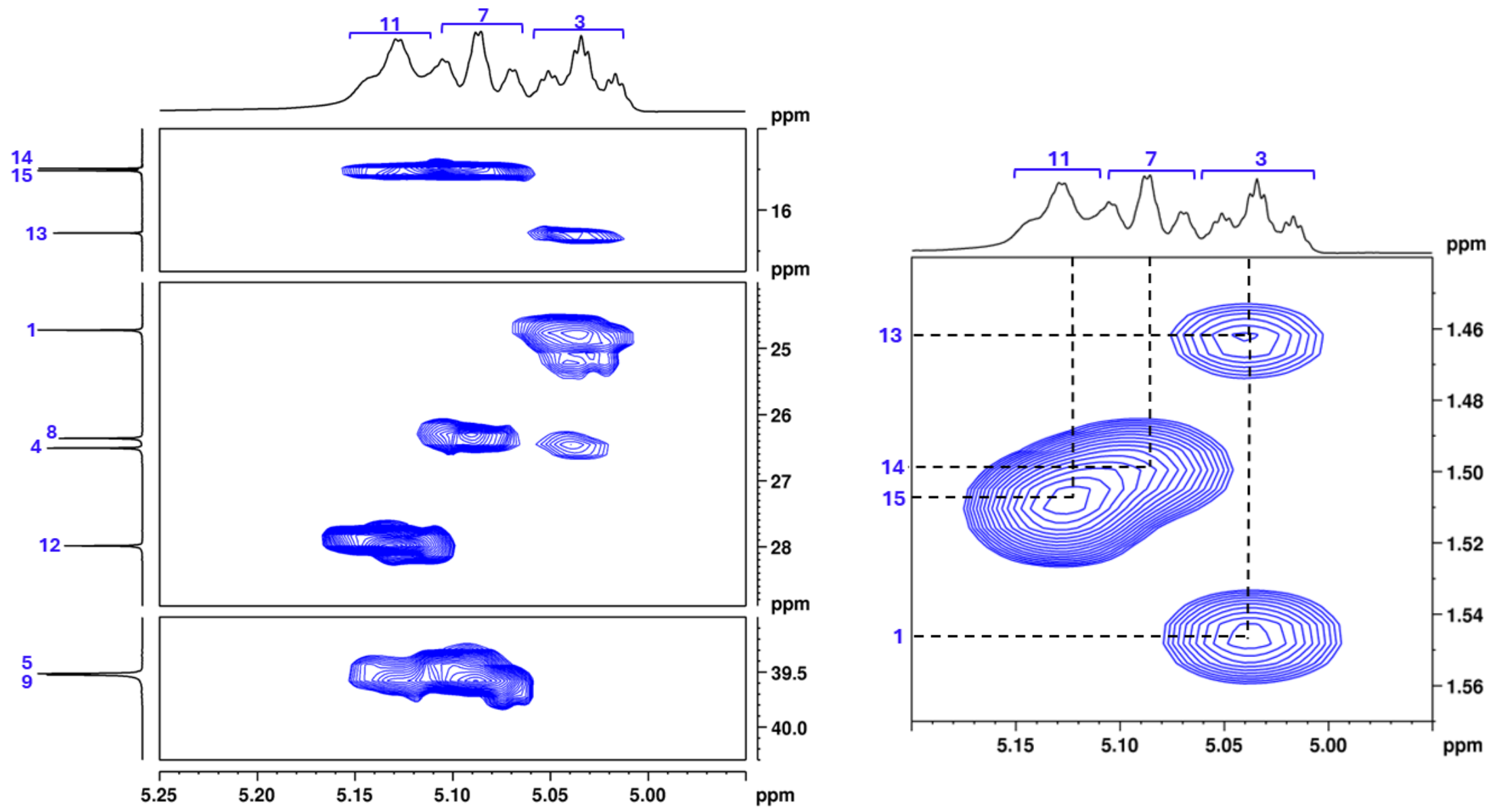

Figure S5. Expansions of the HMBC (left) and ROESY (right) spectra of squalene.

2. NMR Characterization of Squalane obtained by Squalene Hydrogenation

Completely assigned ^{1}H and ^{13}C NMR spectra of squalene obtained by squalene hydrogenation with catalyst Ir-1-RR are reported in **Figure S6**. 2D NMR experiments allowed to assign all resonances, as explained below. This assignment, reported as an example for squalane obtained with Ir-1-RR, agrees with those previously reported in literature for squalane.[1]

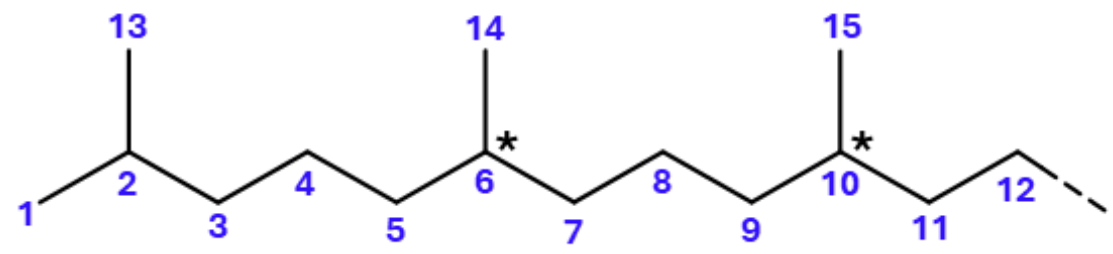

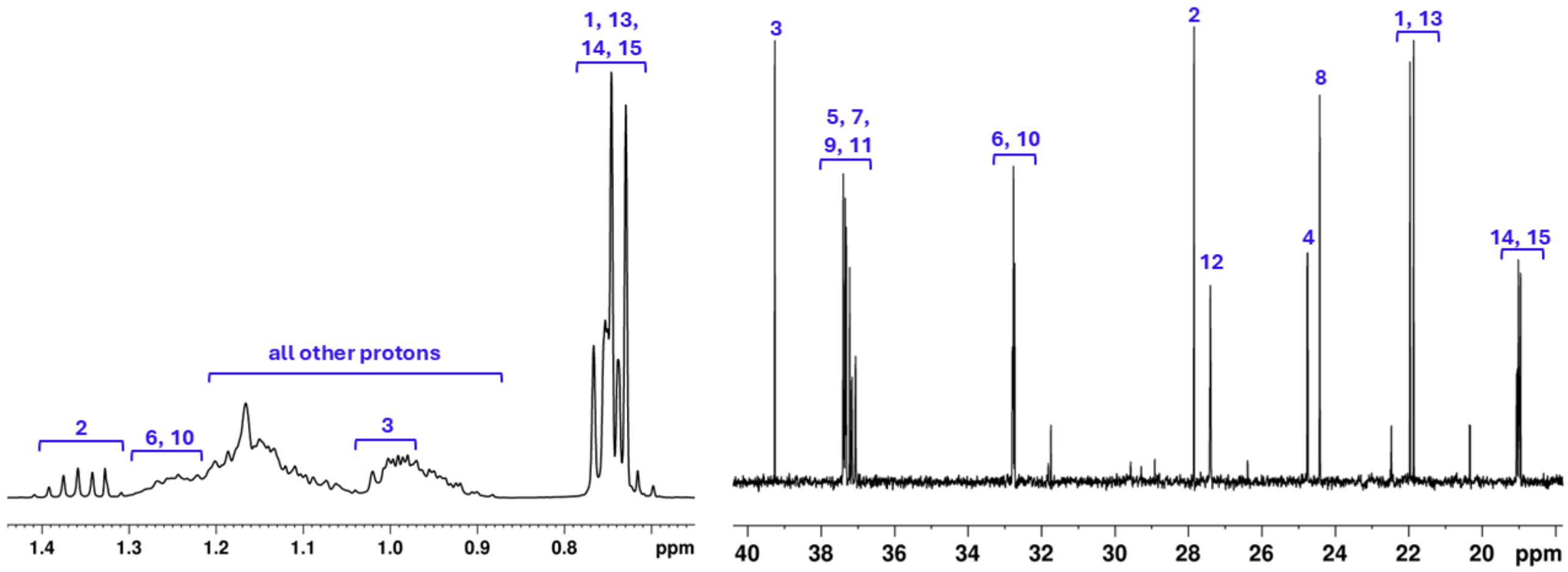

Figure S6. ^{1}H (left) and ^{13}C (right) NMR spectra of squalane.

From the HSQC experiment (**Figure S7**), we identified the two terminal methyl groups (**1** and **13**) resonating at $\delta(^{1}H)$=0.73 ppm and $\delta(^{13}C)$=21.8/9 ppm, and the two internal methyl groups (**14** and **15**) which resonate at $\delta(^{1}H)$=0.75 ppm and $\delta(^{13}C)$=18.9/19.9 ppm. In the ^{13}C NMR spectrum, there are two separate peaks for **1** and **13**, identical in intensity. The COSY experiment (**Figure S7**) shows correlation between the methyl protons at $\delta(^{1}H)$=0.73 ppm and the multiplet at $\delta(^{1}H)$=1.36 ppm, scalarly coupled to $\delta(^{13}C)$=27.8 ppm, which is attributed to **2**, according also to multiplicity. The methyl protons at $\delta(^{1}H)$=0.75 ppm correlate with $\delta(^{1}H)$=1.25 ppm, scalarly coupled to the resonances at $\delta(^{13}C)$=32.7/9 ppm, which are attributed to **6** and **10** instead.

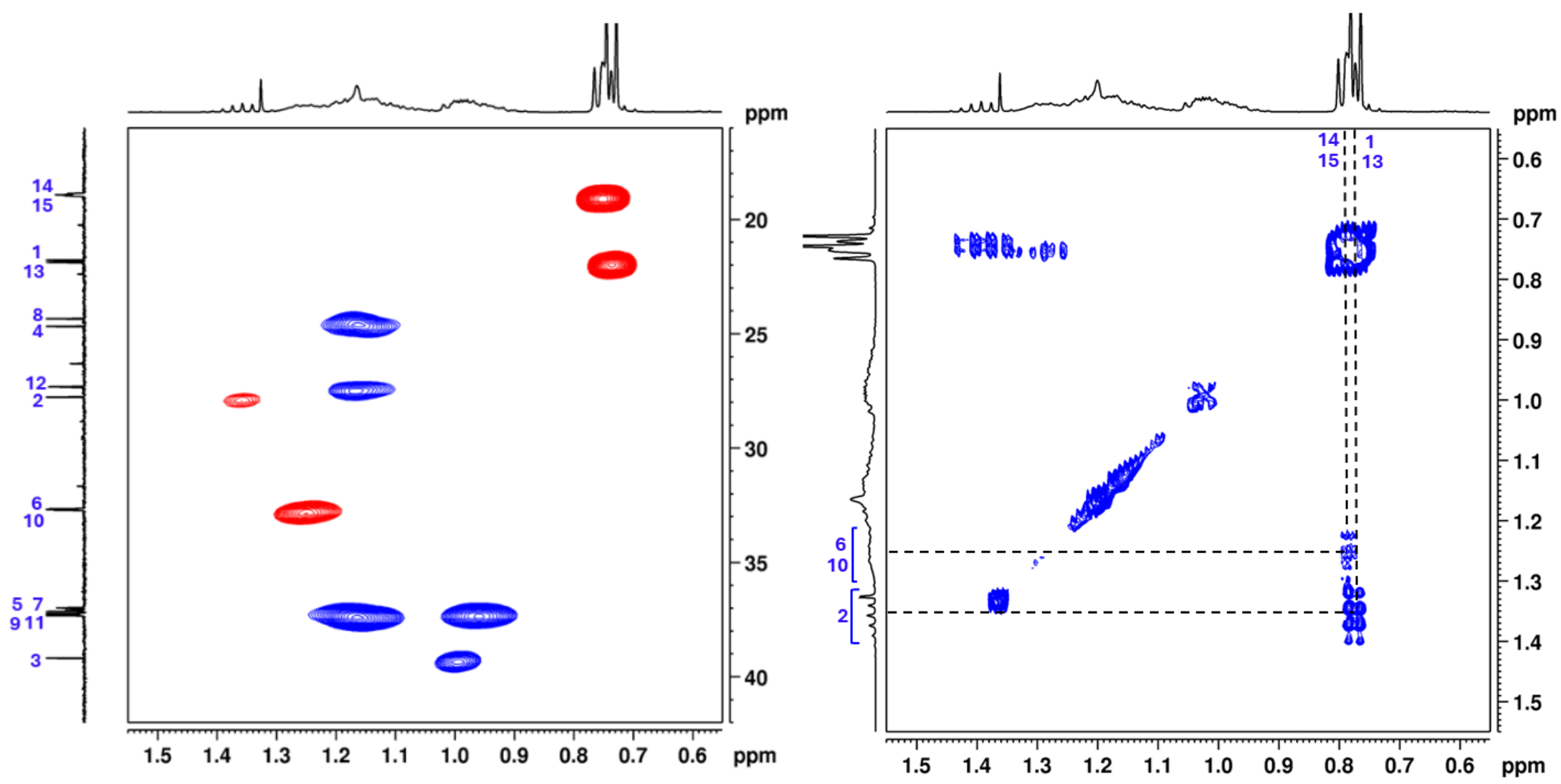

Figure S7. HSQC (left) and COSY (right) NMR spectra of squalane.

The previous assignment is confirmed by the HMBC experiment (**Figure S8**), which additionally show long-range correlations between the methyl groups **1**, **13** and the carbon atoms resonating at $\delta(^{13}C)$=39.3 ppm, attributed to **3**, while the methyl groups

14, **15** correlate to resonances at δ(^{13}C)=37.0/5 ppm, attributed to methylenes **5**, **7**, **9**, **11**, based also on peak integration (x4). As showed in **Figure S8**, the HMBC spectrum helped to identify also **4**, **8** and **12**, because of long-range correlations with **2** and methylenes **5**, **7**, **9**, **11**.

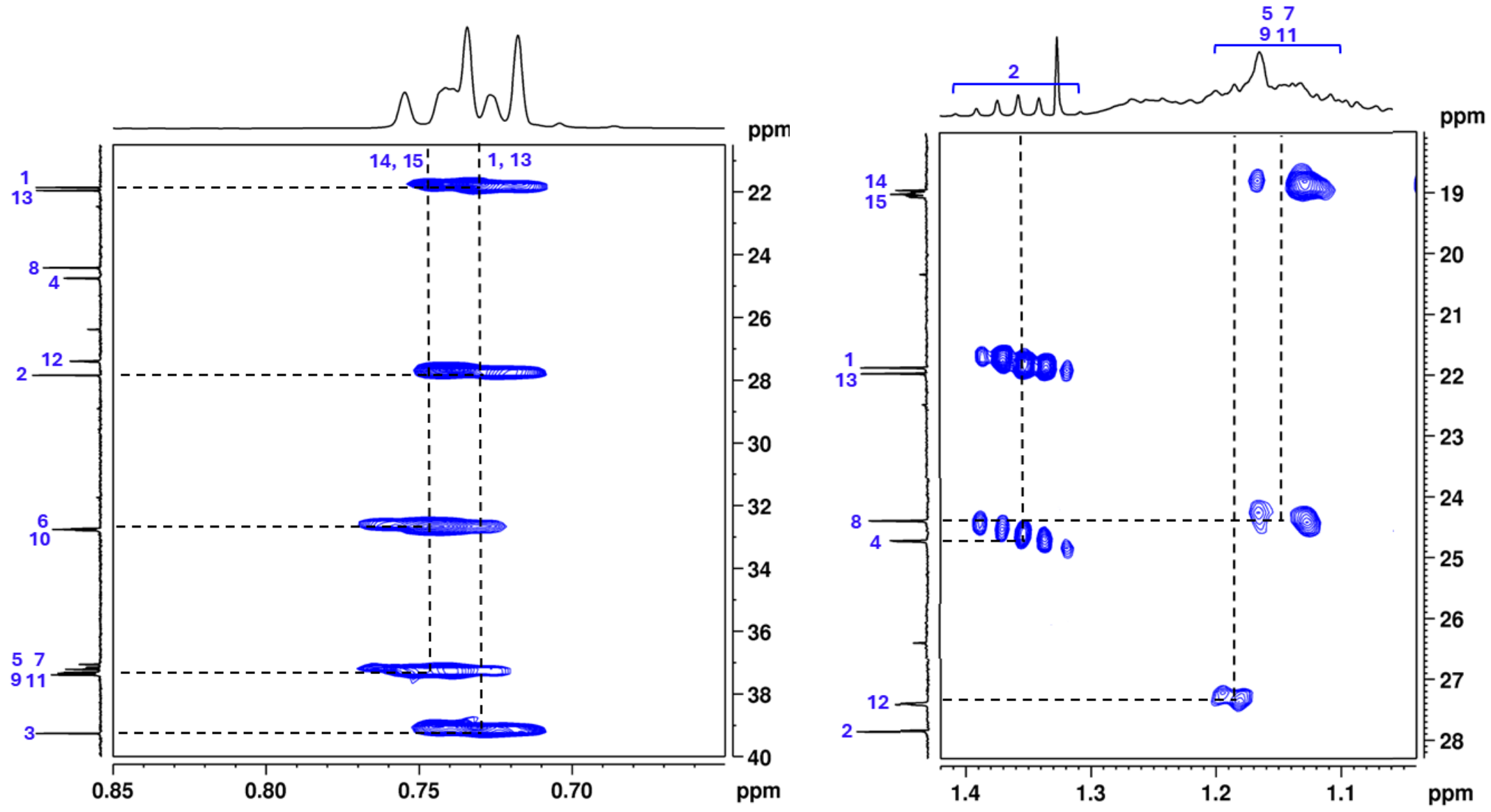

Figure S8. Expansions of the HMBC NMR spectra of squalane.

3. ^{13}C NMR spectra of Hydrogenated Squalene from All Catalysts

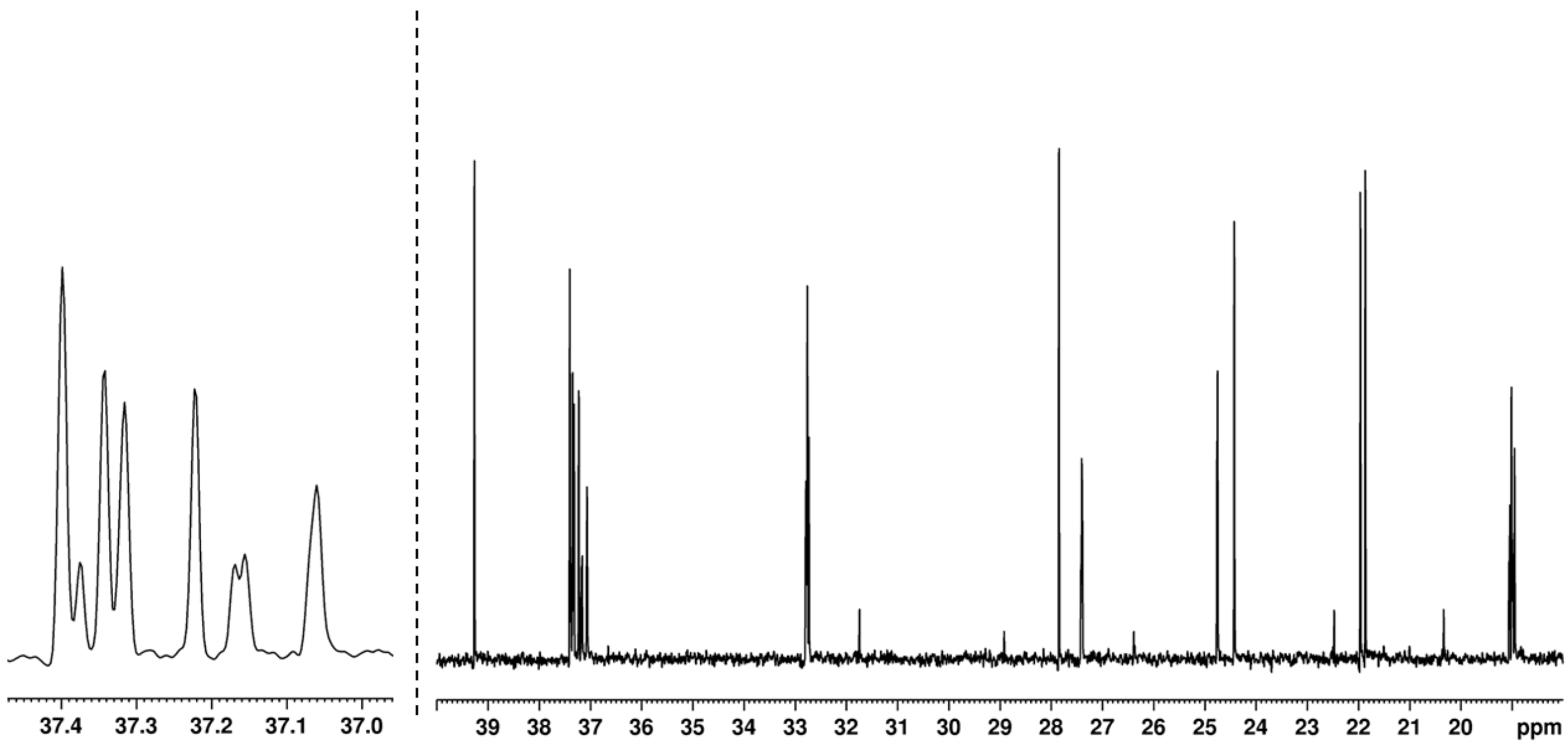

Figure S9. Full (right) ^{13}C NMR spectrum and expansion of methylene region (left) of squalene hydrogenated with Ir-1-*RR* in *o*DFB.

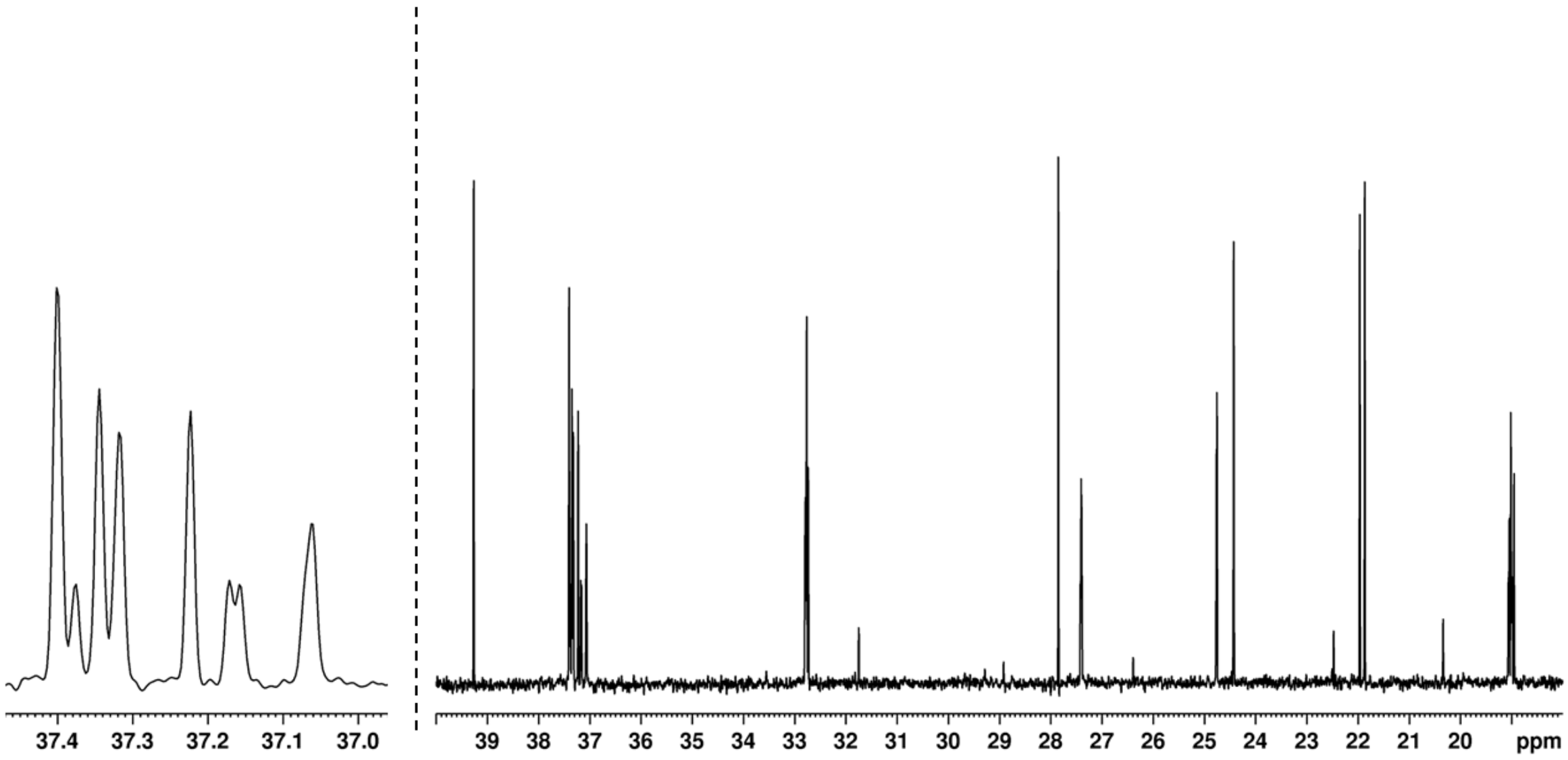

Figure S10. Full (right) ^{13}C NMR spectrum and expansion of methylene region (left) of squalene hydrogenated with Ir-1-*SS* in *o*DFB.

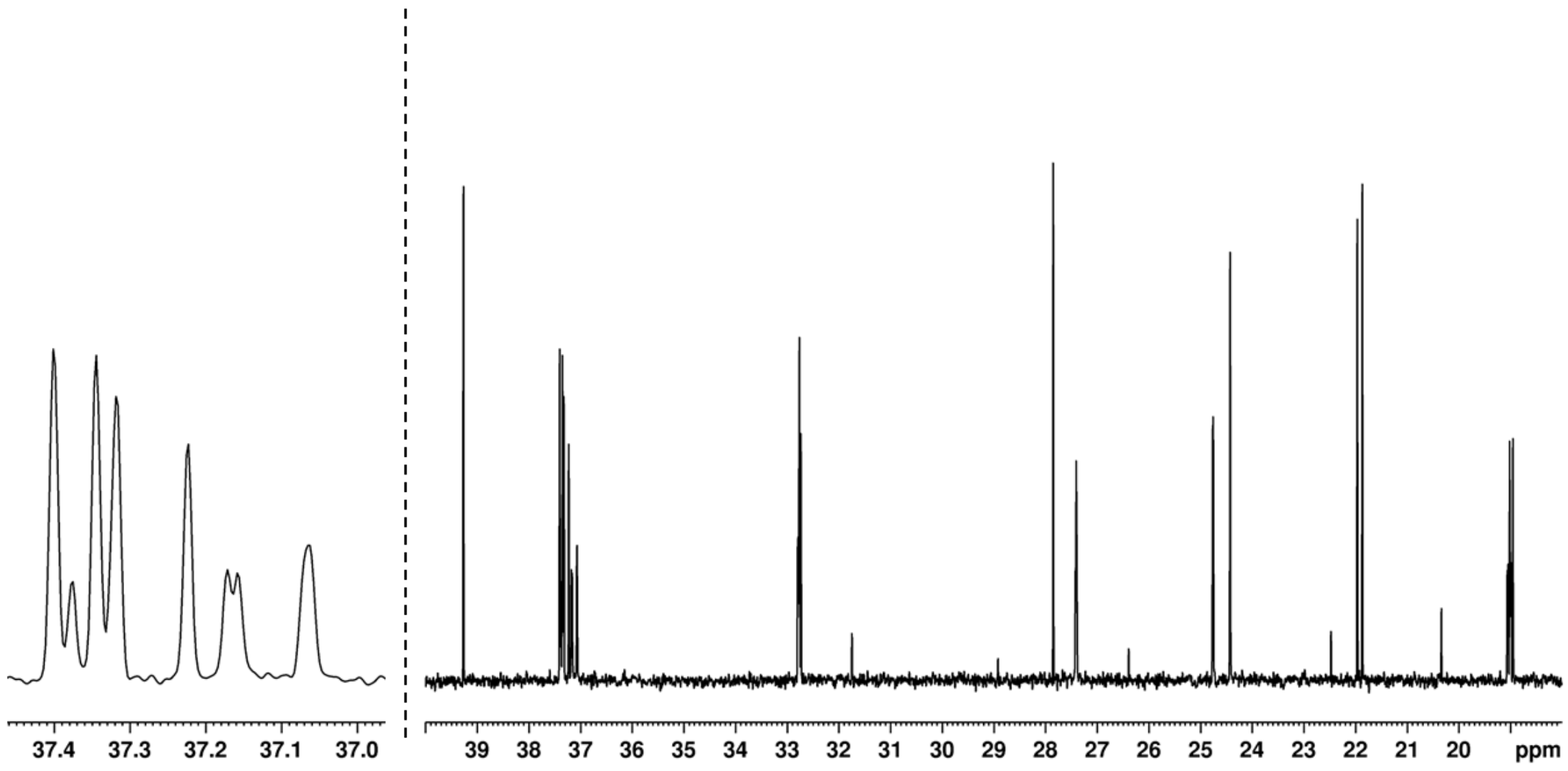

Figure S11. Full (right) ^{13}C NMR spectrum and expansion of methylene region (left) of squalene hydrogenated with Ir-2-*RR* in *o*DFB.

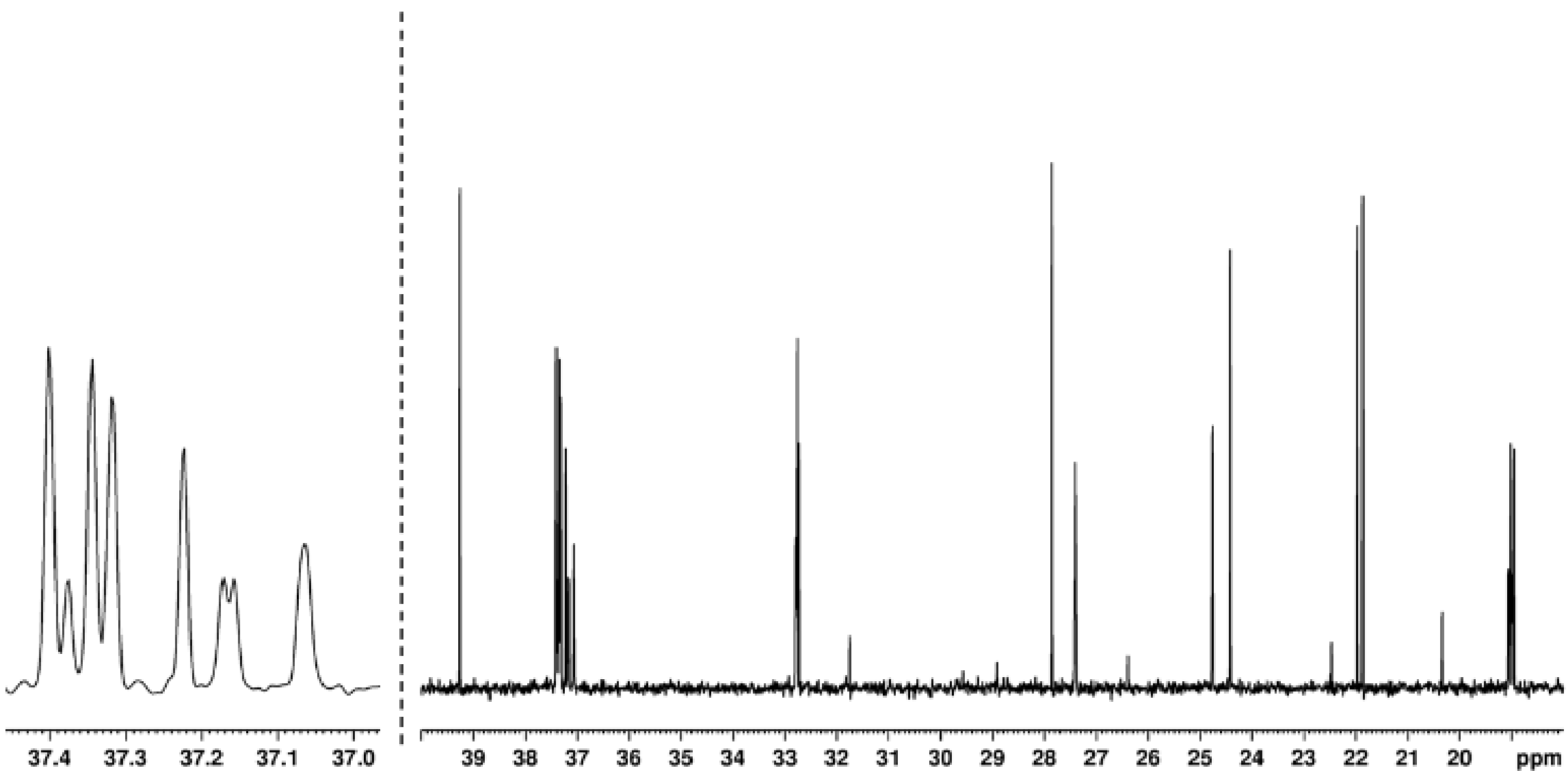

Figure S12. Full (right) ^{13}C NMR spectrum and expansion of methylene region (left) of squalene hydrogenated with Ir-2-*SR* in *o*DFB.

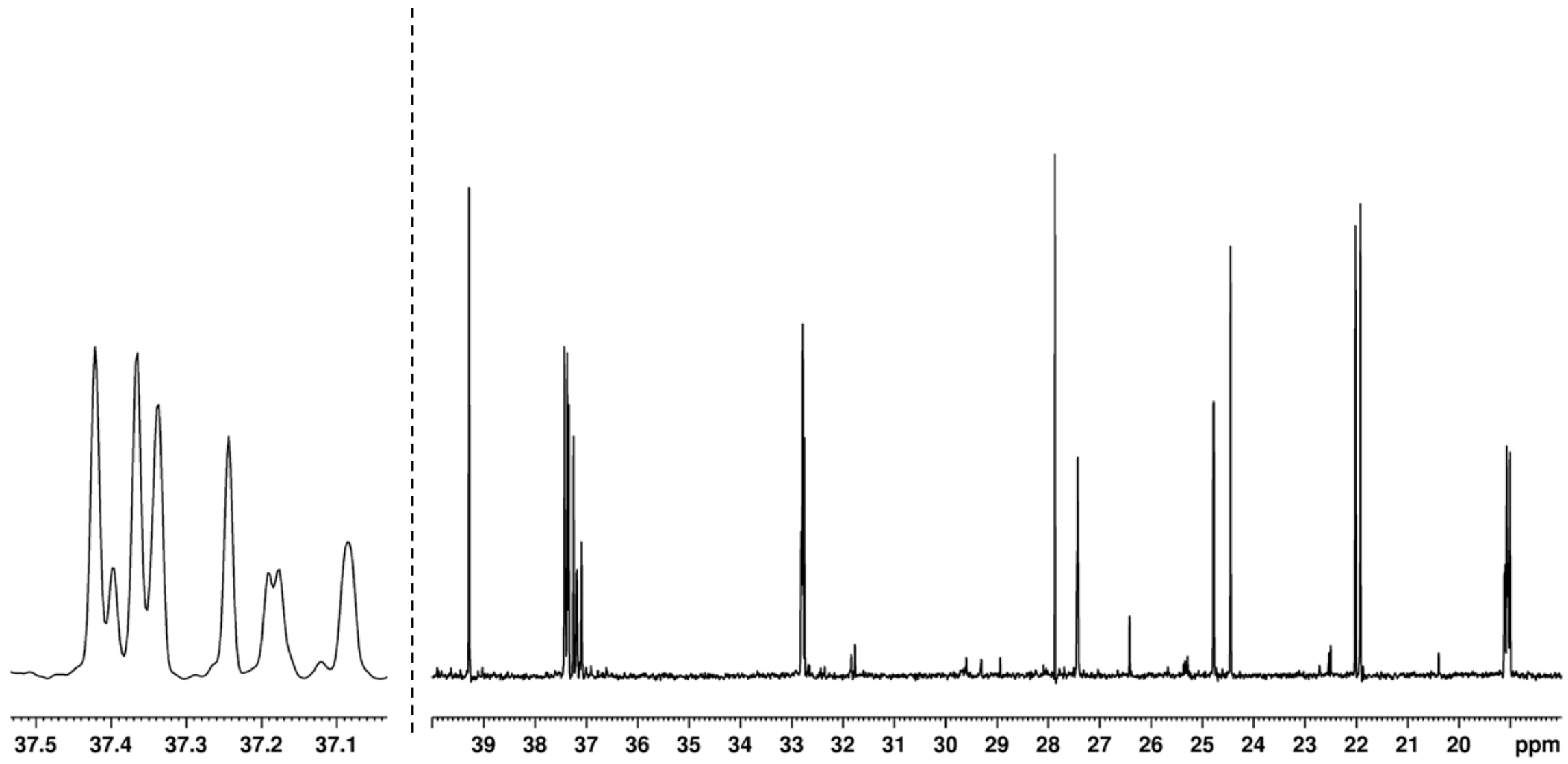

Figure S13. Full (right) ^{13}C NMR spectrum and expansion of methylene region (left) of squalene hydrogenated with Rh-2-*RR* in *o*DFB.

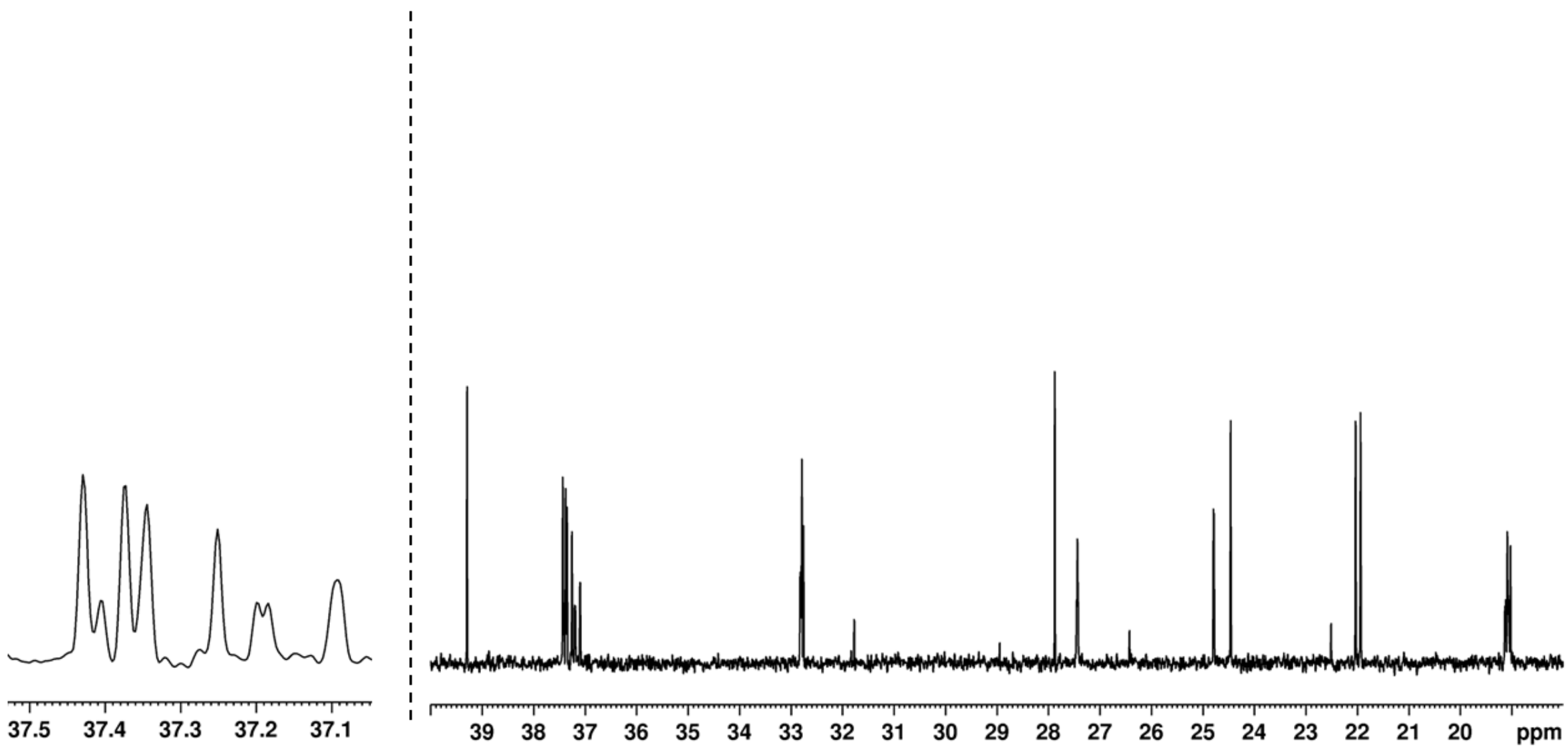

Figure S14. Full (right) ^{13}C NMR spectrum and expansion of methylene region (left) of squalene hydrogenated with Rh-2-*SS* in *o*DFB.

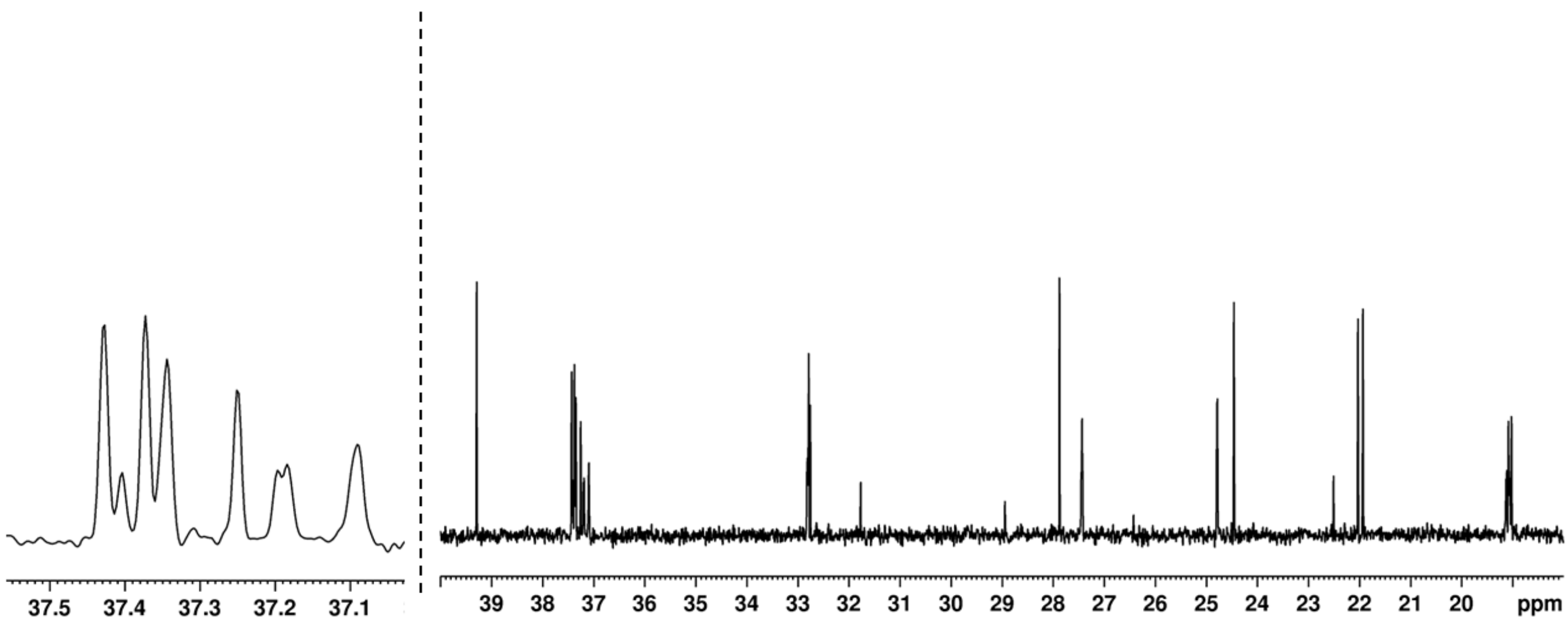

Figure S15. Full (right) ^{13}C NMR spectrum and expansion of methylene region (left) of squalene hydrogenated with Rh-3-*SS* in *o*DFB.

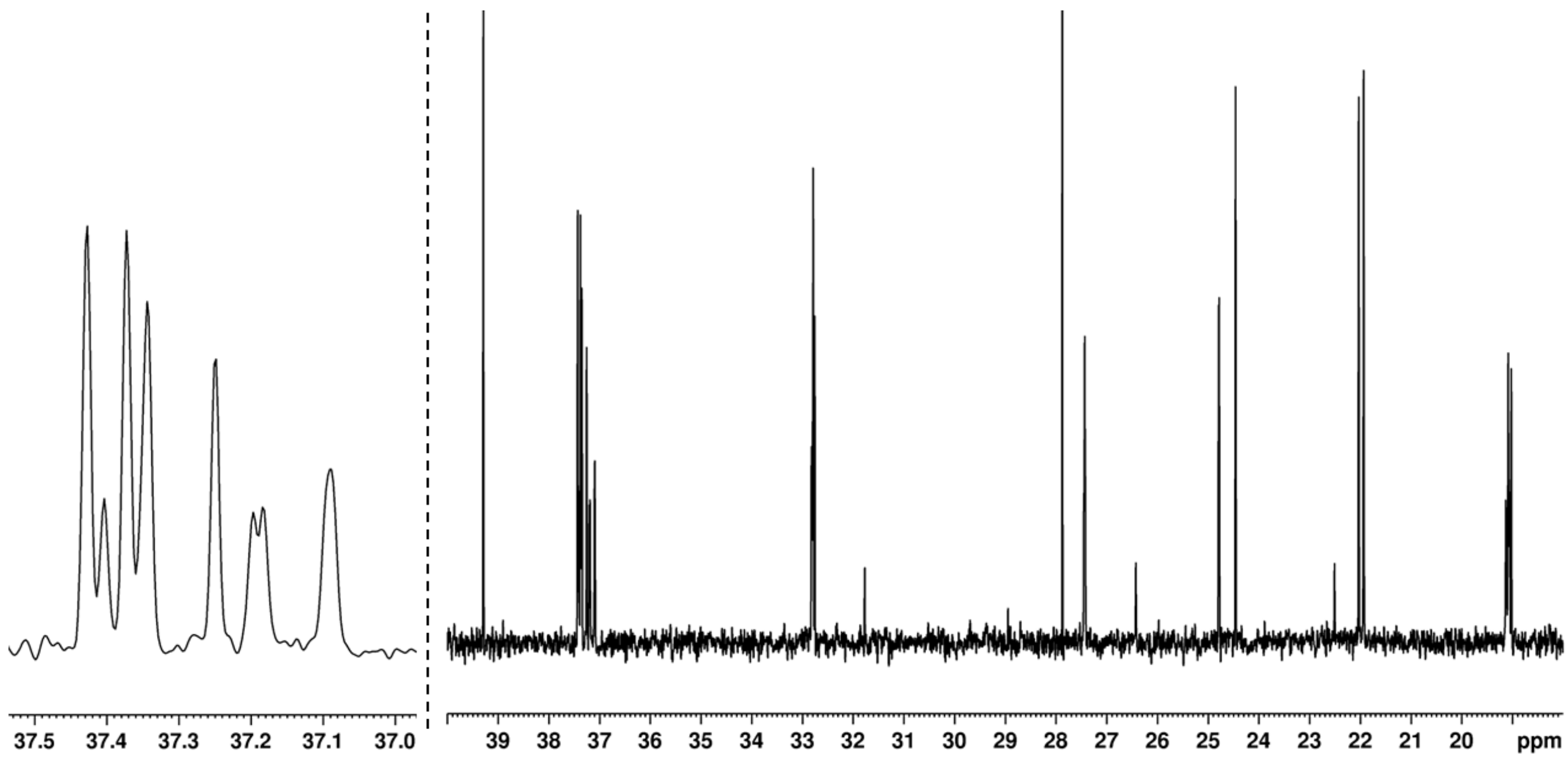

Figure S16. Full (right) ^{13}C NMR spectrum and expansion of methylene region (left) of squalene hydrogenated with Rh-5-*SS* in *o*DFB.

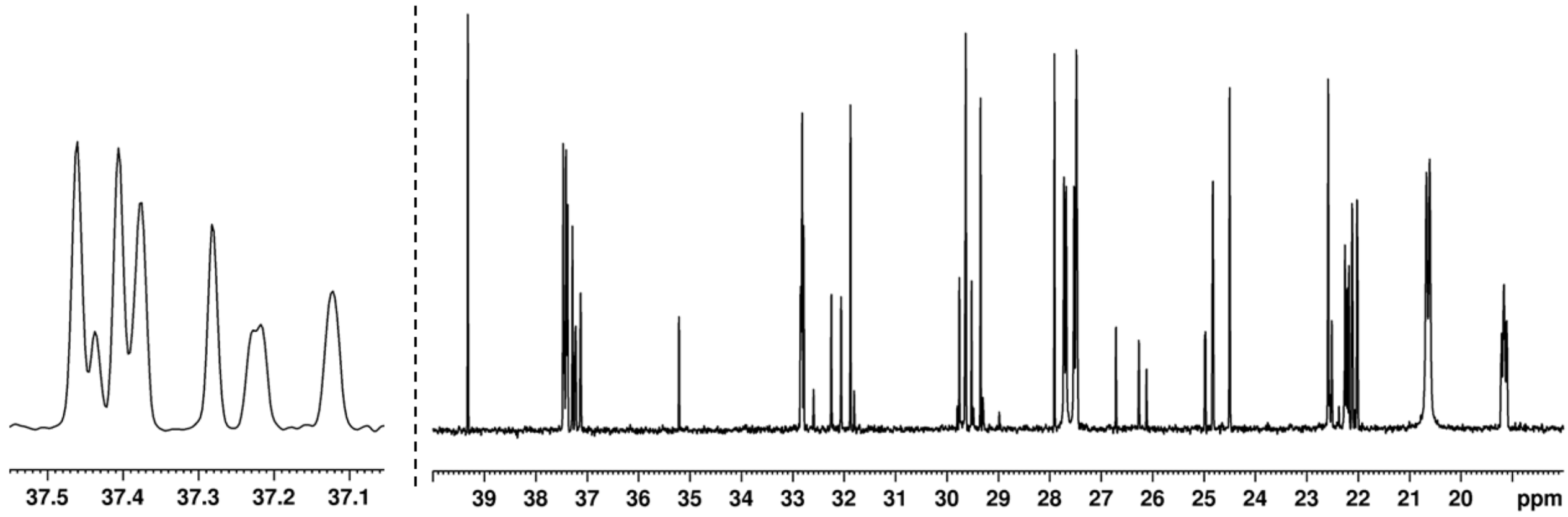

Figure S17. Full (right) ^{13}C NMR spectrum and expansion of methylene region (left) of squalene hydrogenated with Pd/C in *o*DFB.

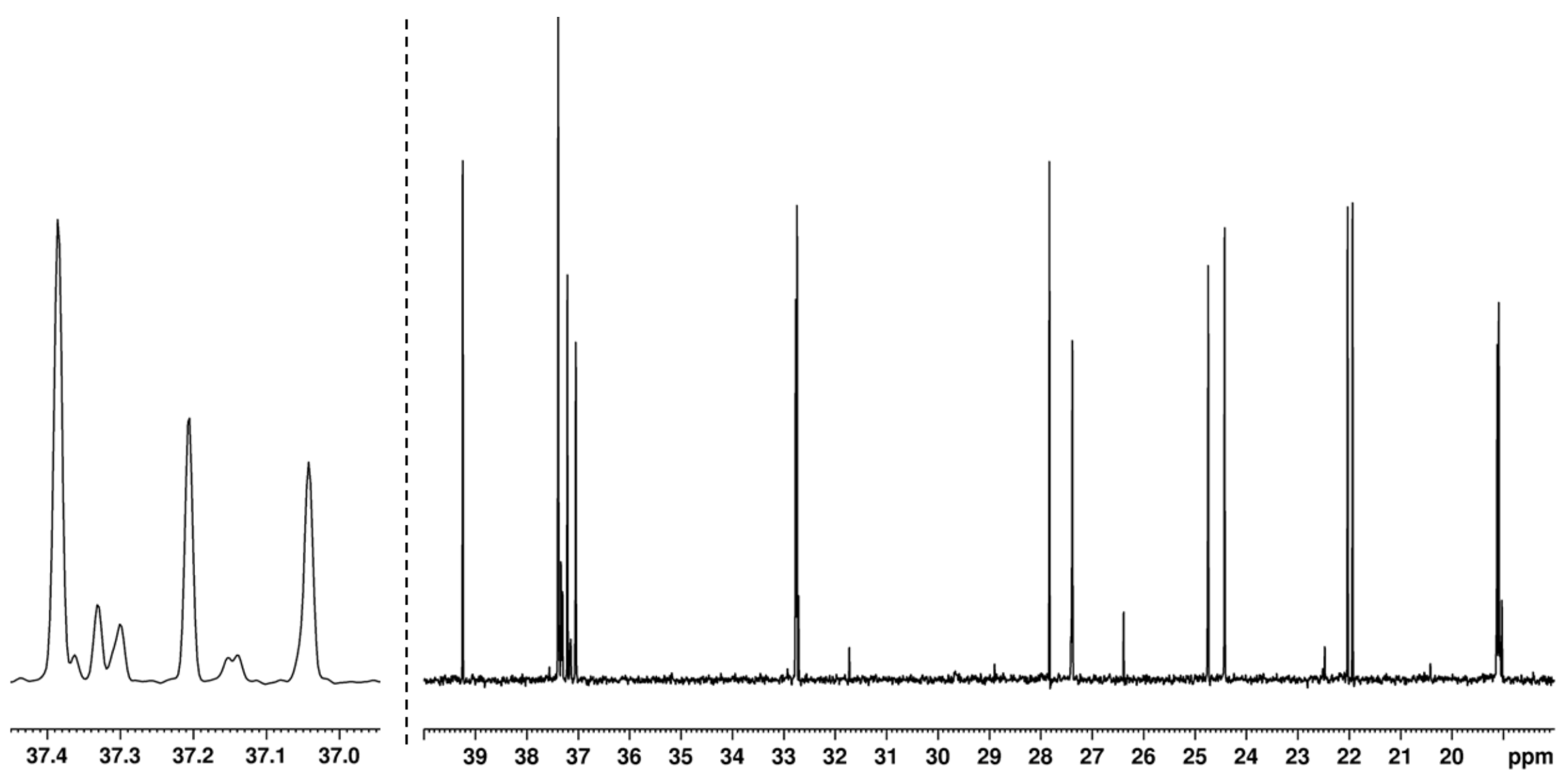

Figure S18. Full (right) ^{13}C NMR spectrum and expansion of methylene region (left) of squalene hydrogenated with Ir-3-*S* in *o*DFB.

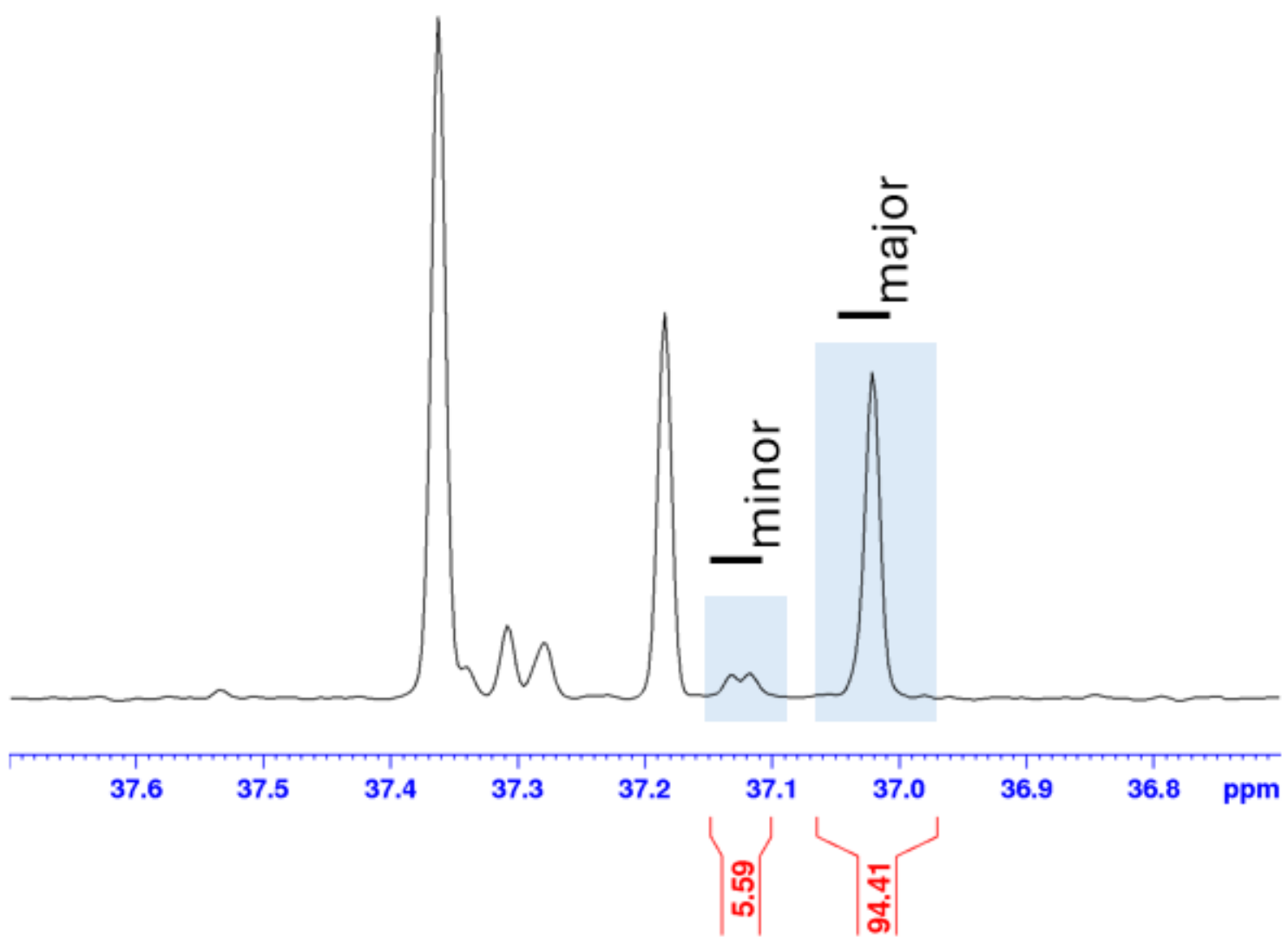

Figure S19. Expansion of methylene region (left) of squalene hydrogenated with Ir-3-*S* in *o*DFB.

The diastereomeric excess (% d.e.) was calculated using the following equation, where I_{major} and I_{minor} are the integrated areas of the C11 resonances assigned to the major and minor diastereomeric populations, respectively:

$$\% \, \mathrm{d.e.} = 100 \times \frac{I_{\mathrm{major}} - I_{\mathrm{minor}}}{I_{\mathrm{major}} + I_{\mathrm{minor}}}$$

The resonances assigned to the major and minor diastereomeric populations were integrated as indicated in Figure S19. For the representative spectrum shown in Figure S19, the normalized integrals were $I_{\mathrm{major}} = 94.4$ and $I_{\mathrm{min}} = 5.6$, corresponding to 89% d.e..

4. Hydrogenation Experiment Data

All hydrogenation efficiencies evaluated from ^{1}H NMR spectra are reported in the following table. Ir-catalysts and Pd/C showed 100% hydrogenation efficiency for each experiment and are not reported.

Table 1. Hydrogenation Efficiencies of Rh-catalysts.

CATALYST	Hydrogenation efficiency %	Average %
Rh-1-*RR*	65	71
	75	
	73	
Rh-1-*SS*	22	23
	20	
	26	
Rh-2-*RR*	98	98
	99	
	96	
Rh-2-*SS*	94	95
	96	
	95	
Rh-3-*RR*	25	28
	26	
	31	
Rh-3-*SS*	98	98
	97	
	98	
Rh-4-*RR*	51	52
	48	
	56	
Rh-4-*SS*	73	73
	71	
	74	
Rh-5-*RR*	95	94
	90	
	98	
Rh-5-*SS*	98	98
	98	
	98	
